# (Re)building Cooperation: Effects of a Cognitive Intervention on Cooperative Behaviour in Games

**DOI:** 10.5334/cpsy.178

**Published:** 2026-09-16

**Authors:** Ismail Guennouni, Samuel Dupret, Quentin J. M. Huys, Maarten Speekenbrink

**Affiliations:** 1 Department of Experimental Psychology, Division of Psychology and Language Sciences, UCL, UK; 2 Applied Computational Psychiatry Lab, Mental Health Neuroscience Department, Division of Psychiatry and Max Planck UCL Centre for Computational Psychiatry and Ageing Research, Department of Imaging Neuroscience, Queen Square Institute of Neurology, UCL, UK

**Keywords:** Social Trust, Repair of Cooperation, Cognitive Intervention, Economic Games, Hidden Markov Models

## Abstract

Cooperation often breaks down when one party appears to withdraw trust, yet most research has focused on initial trust formation rather than how the aggrieved party can resist the impulse to retaliate. In a randomised controlled online trial (N = 318), we tested whether a brief cognitive intervention inspired by Dialectical Behaviour Therapy principles could promote resilient cooperation in the face of trust withdrawals. Participants played a Repeated Trust Game as trustees against adaptive computerised investors driven by hidden Markov models (HMMs) trained on human data, providing behavioural realism under full experimental control. Following the intervention, participants returned a significantly higher proportion of the amount received than an active control group across the post-manipulation game. The groups diverged most sharply at the pre-programmed low investment, where the control group retaliated by reducing returns while the intervention group maintained the same level of returns. This shift occurred without a change in self-reported emotional reactivity, dissociating the preserved emotional reaction from the retaliatory behaviour it drives. HMM analysis of participants’ own return patterns revealed that participants in the intervention condition were less likely to become stuck in low-return behavioural states following the pre-programmed low investment, though a notable minority did not respond to the intervention. The intervention effect did not transfer to a Prisoner’s Dilemma game, consistent with context-specific effects. These findings provide proof-of-concept evidence that targeted cognitive strategies can support cooperation after trust violations, and show that HMM-based agents are a useful tool for studying social behaviour under controlled conditions.

## Introduction

Cooperation is fundamental to collective success and social harmony (Tomasello et al., 2012). At the heart of cooperation is trust, the belief that others will act in mutually beneficial ways even when they could exploit the situation (Rousseau et al., 1998). Trust enables risky interactions where immediate self-interest could override long-term benefits (Balliet & Van Lange, 2013). Without trust, cooperation tends to break down, leading to suboptimal outcomes for all parties involved. We ask how the aggrieved party in such a breakdown can resist the impulse to retaliate, and whether a brief cognitive intervention can help them sustain cooperation.

The Repeated Trust Game (RTG) has emerged as a well-established paradigm for studying trust and cooperation in controlled settings (Berg et al., 1995). In this game, an “investor” decides how much of an endowment to send to a “trustee.” The amount sent is typically multiplied by 3, and the trustee then decides how much of this multiplied amount to return to the investor and how much to keep for themselves. Cooperation emerges when both parties act for mutual gain, but trust is fragile: a single failure to reciprocate can trigger a breakdown (Bendor et al., 1991) that is difficult to repair even when repair would be mutually beneficial (Harth & Regner, 2017).

Previous research has explored ways to encourage initial cooperation in trust games, such as using third-party enforcement (Charness et al., 2008; Fiedler & Haruvy, 2017) and priming with concepts of gratitude (Drążkowski et al., 2017) or friend and foe (Burnham et al., 2000). While these approaches increase early cooperation, they often fail to address the more challenging task of repairing cooperation after trust has been broken. The trust repair literature has examined what transgressors can do (apologies, promises, penance) to restore damaged relationships (Bottom et al., 2002; Kim et al., 2004; Schweitzer et al., 2006; Tomlinson & Mayer, 2009). Yet the recipient’s side of this equation has received less attention: how the aggrieved party regulates the impulse to retaliate when continued cooperation would serve them better.

We focus on the role of the trustee in the RTG in establishing and maintaining cooperation. As trustees, participants do not extend trust under uncertainty as the investor does; they respond to the trust extended or withdrawn. Their decision is one of reciprocity, of whether to retaliate or to restore the relationship after a breach, and this response sustains or erodes cooperation. A lack of trustee reciprocation erodes trust over time, particularly in borderline personality disorder (Lieb et al., 2004). These trustees fail to engage in trust-repairing behaviours such as coaxing the investor by signalling trustworthiness via sending high returns (King-Casas et al., 2008). Coaxing behaviour appears to depend on the trustee’s ability to correctly infer the investor’s latent trust state (Guennouni et al., 2024). Unpredictable behaviour from trustees fosters mistrust and impedes future cooperation (Rigdon et al., 2007). In contrast, consistent cooperation from trustees promotes trust and encourages further collaboration, as evidenced by neural data (King-Casas et al., 2005).

We therefore ask whether an intervention targeting interpersonal decision-making can influence trustee behaviour. Dialectical Behaviour Therapy [DBT; Linehan (1993)] offers relevant principles for this purpose. DBT’s distress tolerance skills help individuals withstand negative emotions without acting impulsively, while the “opposite action” technique encourages acting contrary to emotion-driven urges when those urges are counterproductive (Linehan, 2015). When an investor withdraws trust by sending a low investment, the trustee’s natural impulse is often to retaliate; yet this impulse, if acted upon, can trap both parties in a spiral of declining cooperation. An intervention that helps trustees pause, evaluate whether retaliation serves their long-term interests, and consider alternative responses could interrupt this cycle. We test, in a randomised controlled trial, a brief multi-component cognitive intervention inspired by DBT principles, combining reflection on long-term consequences with prosocial guidance, as is typical of real-world cognitive interventions (Linehan, 2015). We hypothesise that prompting reflection on long-term consequences and non-impulsive responses leads to more resilient cooperation after perceived non-cooperation.

This study makes three contributions. First, we address the underexplored recipient side of trust repair: how the aggrieved party regulates the impulse to retaliate when continued cooperation would serve them better. Second, we introduce hidden Markov model (HMM)-based computerised agents trained on human data. Unlike fixed computer strategies used in previous economic game studies, these agents produce adaptive, probabilistic responses driven by a latent “trust state” that reacts to participants’ returns, combining experimental control with behavioural realism. Third, we examine behavioural change not only through traditional aggregate measures but also through HMM analysis of participants’ own response patterns, revealing individual differences in susceptibility to becoming “stuck” in low-cooperation states following the pre-programmed low investment.

We conducted an online experiment with 318 participants acting as trustees in two 15-round RTGs, with the intervention administered between games to a subset of participants. To foreshadow our results, the intervention raised returns across the post-manipulation game and prevented the drop in returns that the control group showed at the pre-programmed low investment. The effect held whether or not participants believed they were facing a human, and it did not transfer to a Repeated Prisoner’s Dilemma.

## Methods

### Participants and design

**Figure 1:**
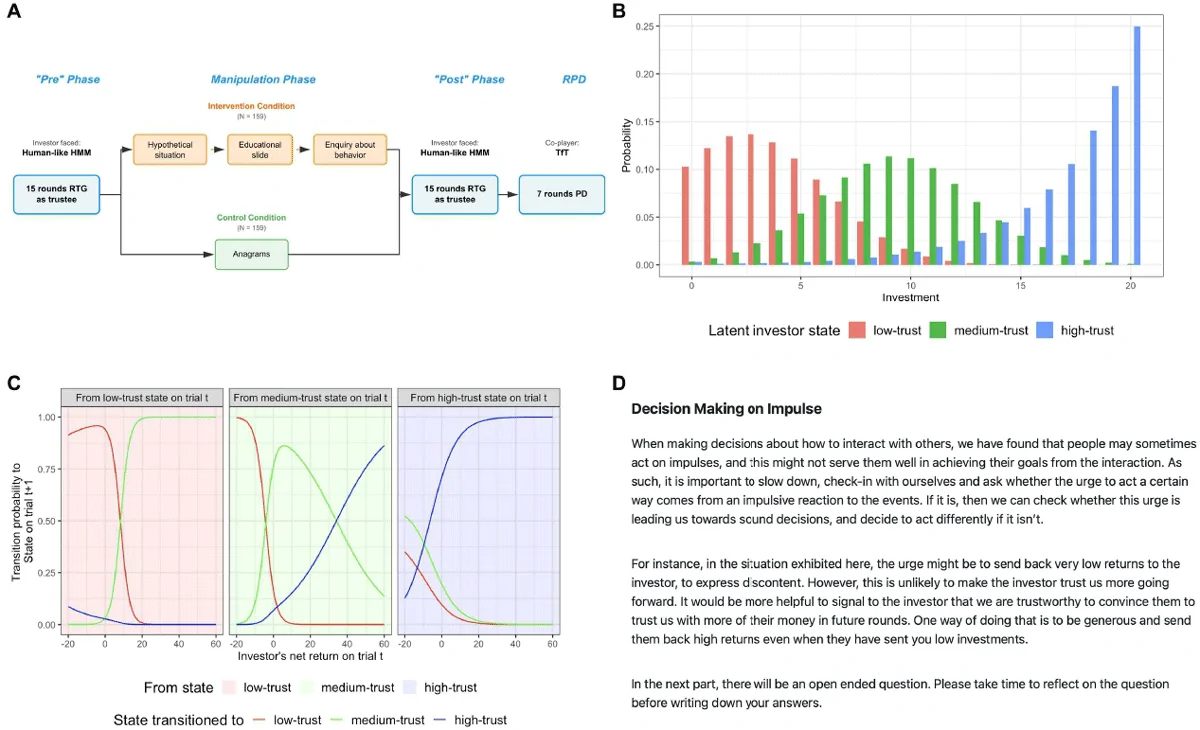
**Panel A:** Experimental design overview. Participants in both conditions played two 15-round Repeated Trust Games (RTGs) with human-like hidden Markov model (HMM) investors, separated by a manipulation phase. The intervention condition received a cognitive intervention, while the control condition solved anagrams. Both groups then completed a 7-round Repeated Prisoner’s Dilemma (RPD) with a tit-for-tat (TFT) co-player to assess transfer effects. This design allows for comparison of cooperative behaviour before and after the manipulation, as well as between conditions. **Panels B–C:** We constructed the artificial investor agent by fitting a three-state hidden Markov model to data from human investors engaged in the 10-round Repeated Trust Game against human trustees. From the fitted HMM, we obtained the distribution of investments by the artificial investor agent conditional on its latent state as shown in Panel B. The fitted HMM also yields the state-transition probabilities as a function of the net return (difference between the investment sent and the amount received in return) on trial t as shown in Panel C. Each plot in Panel C represents a different starting latent state on trial t, and each line represents the probability of transitioning to a particular state in trial t+1. **Panel D:** Full text of the educational slide shown to the intervention group.

The experiment employed a 2 (Condition: Intervention or Control) by 2 (Game: Trust-Game Pre-Manipulation, Trust-Game Post-Manipulation) design, with repeated measures on the second factor. The experimental procedure followed a fixed sequence: RTG Phase 1 (15 rounds as trustee) → Intervention or Control manipulation → RTG Phase 2 (15 rounds as trustee) → Repeated Prisoner’s Dilemma (7 rounds) → Post-game questionnaires (see Figure 1A for a graphical overview). A total of 320 participants were recruited on the Prolific Academic platform (prolific.co). The study was not pre-registered. The required sample size was set by an a priori power analysis with G*Power (Faul et al., 2009) (power .8, α = .05) to detect a small within-between interaction (Cohen’s f = 0.10) in a 2 × 2 mixed ANOVA: the omnibus Condition × Game interaction on returns across the post-manipulation game, which is our primary confirmatory test. The event-study around the pre-programmed low investment, where the divergence is sharpest, is a focused secondary analysis that examines this divergence and was not powered separately. Further secondary analyses are the HMM state analysis, emotion self-reports, the investor-rating appraisal, transfer to the Prisoner’s Dilemma, and the belief-subgroup analysis. Participants were randomly assigned to either the intervention or control condition. Two players had incomplete trust game entries and their data was disregarded, leaving data from 318 participants for analysis, equally split between the two conditions. The mean age of participants was 31.3 years (SD = 9.9). Participants were paid a fixed fee of £5 plus a performance-contingent bonus payment of £0.71 on average (see Ethics and Consent).

### Tasks and Measures

#### Repeated Trust Game

Participants played two separate 15-round Repeated Trust Games (Berg et al., 1995) in the role of trustee. In each round of the RTG, the (computer-simulated) investor is endowed with 20 units and decides how much of that endowment to invest. This investment is tripled, and the trustee (participant) then decides how to split this tripled amount between themselves and the investor. If the trustee returns more than one third of the amount they receive, the investor makes a gain.

On each round, immediately after being informed of the investment sent, participants made a two-dimensional rating by clicking on a labelled grid (see supplement for a screenshot). The intervention group rated their emotion (valence from negative to positive, arousal from low to high). The control group rated the investment (speed from slow to fast, magnitude from low to high); this is not an emotion measure. Both conditions thus had a matched per-round grid task. These per-round responses are separate from the four post-game investor ratings described below.

The strategy of the computerised investor was modelled on behaviour of human investors in a 10-round Repeated Trust Game (RTG) with the same co-player. Details of the data sources used for this are provided in the Supplement (Section 6). Using this data, we estimated a hidden Markov model (HMM) for investors’ behaviour with three latent states (Guennouni et al., 2024). Each latent state was associated with a state-conditional distribution over the possible investments from 0 to 20 (Figure 1B). These distributions reflect “low-trust”, “medium-trust”, or “high-trust”. Over rounds, the investor can switch state, and the probability of such state transitions is a function of the net return (i.e. return - investment) in the previous round (see Figure 1C). To probe trust repair, the agent was programmed to send a low investment on round 12 (pre-manipulation) or round 13 (post-manipulation). On all other rounds, investments were drawn from the state-conditional distribution, and state transitions followed the net return on the previous round. The transition into the trial immediately after each pre-programmed low investment was the one exception. That low investment was experimentally imposed, not produced by the agent’s own state, so its net return was not used to update the agent’s state. The forced low investment therefore did not distort the agent’s behaviour on the rounds that followed. The HMM investor began each game in the “medium-trust” state.

#### Intervention

The intervention was inspired by Dialectical Behaviour Therapy [DBT; Linehan (2015)] skills training. Within DBT’s distress-tolerance and emotion-regulation modules, it instantiates the opposite-action skill (acting contrary to an emotion-driven urge when that urge is counterproductive), delivered as brief psychoeducation about long-term consequences rather than as in-session skills practice. We did not implement mindfulness practice or full emotion-regulation skill rehearsal, and the design cannot dissociate the opposite-action framing from the explicit psychoeducational instruction. Specifically, participants were presented with a hypothetical situation in which they receive a low investment and asked to indicate how they would respond. They were then shown an educational slide (its full text appears in Figure 1D) inviting them to consider the game’s reward-maximising goal and whether punishing the low investment served it. Participants were told that punishment can create a negative feedback loop where the other player might trust them even less. An alternative action was suggested, whereby players would respond kindly to such a transgression in the hope of gaining trust from the investor. Participants were then asked whether the information just received would change their behaviour in such a hypothetical situation and to justify their answer. Each participant’s free-text reply was coded by a large language model (Claude Opus 4.7, Anthropic) in two independent passes against a fixed codebook, the second blind to the first (Cohen’s κ = .90, 95.6% agreement); a co-author adjudicated the disagreements, and the full codebook and per-pass codes are in the Supplement (Section 9.6).

As an active control matched on time-on-task, participants solved five anagrams (“listen”, “triangle”, “deductions”, “players”, “care”) in a free-form text box.

#### Repeated Prisoner’s Dilemma

To ascertain whether any effect of the intervention would transfer to a different game, participants played 7 rounds of a Repeated Prisoner’s Dilemma (RPD). In each round, participants could choose between a cooperative action with a reward of 5 (the other player also cooperates) or 1 (the other player defects), or a defect action with a reward of 7 (the other player cooperates) or 2 (the other player defects). The Nash equilibrium for a single-round version is to choose the non-cooperative action. In the repeated version, both players can maximise their reward by choosing to cooperate. Unlike the Trust Game, where the trustee responds after seeing the investor’s move, a Prisoner’s Dilemma trial requires a simultaneous choice.

In this game, the computerised agent followed a tit-for-tat strategy (Axelrod & Hamilton, 1981): cooperate first, then mirror the participant’s previous action. On round 4 it was pre-programmed to defect regardless of the participant’s preceding action.

#### Post game questionnaires

Failure to repair a breakdown in trust in the repeated trust game has been associated with trustees with BPD traits (King-Casas et al., 2008). Theories of social dysfunction in BPD have focused on dysfunction in the patients’ mentalising ability (Allen & Fonagy, 2006) as well as difficulties in emotional regulation (Rudge et al., 2020). The questionnaires we included in the experiment tried to assess borderline traits (PAI-BOR; Morey (1991)), emotional regulation capabilities (DERS; Gratz & Roemer (2004)) and mentalising ability (RFQ8; Fonagy et al. (2016)). We had directional a-priori expectations that these scores might moderate the intervention effect, although the study was not pre-registered. From the borderline-personality literature on rupture and repair, we expected that higher borderline traits, greater emotion-regulation difficulty, or lower mentalising would, if anything, weaken the behavioural effect of the intervention or increase emotional reactivity to the low investment. The Supplement reports these moderation tests and their pre-specified direction.

### Procedure

At the start of the experiment, participants provided informed consent and were instructed the study would consist of three phases. Participants were led to believe they were interacting with human co-players. They were told they would face the same co-player within all rounds of a game, but that the co-player would change between games (i.e. from the first to the second Repeated Trust Game, and then to the Repeated Prisoner’s Dilemma). Both conditions were told to maximise their total points across all phases, and passed comprehension checks on the number of phases and on the co-player arrangement just described; they were not told the number of rounds in each phase.

Phase one was a 15-round RTG with the participant as trustee, facing the same investor throughout. After detailed game instructions, a further check confirmed they understood their own and the co-player’s payoffs in a hypothetical exchange. On each round they made the grid rating described above, then chose how much of the tripled investment to return; after the 15 rounds they rated the investor on cooperativeness, selfishness, trustworthiness, and friendliness (each from 1 to 10). The intervention group then completed the intervention and the control group solved anagrams, after which phase two repeated phase one, now with a co-player introduced as new.

Phase three consisted of 7 rounds of the Repeated Prisoner’s Dilemma game (RPD), with participants informed they would face a third co-player. Participants then completed questionnaires related to mentalising abilities, emotion regulation, and BPD traits (see the supplement for details). They were then asked about the strategy in the games, as well as whether they thought the other players were human or computer agents. Finally, participants were debriefed and thanked for their participation.

### Statistical analysis

#### Analysis overview

We report the analyses in five steps. First, we test whether the intervention changed the response to the pre-programmed low investment, with an event-study. Second, we test whether that change extended to the rest of the post-manipulation game, using a mixed-effects model of returns across all rounds. Third, we ask where the intervention acts: on emotional reactivity, on social appraisal (the post-game investor ratings), or on the return strategy. Fourth, we test whether the effect transfers to a structurally different game, the Repeated Prisoner’s Dilemma. Fifth, we use hidden Markov models to describe the latent return states and how much participants differ. The variables are recorded at three levels: the individual, the game (pre- and post-manipulation), and the trial. The Supplement lists every variable at each level with its descriptive statistics.

#### Response at the pre-programmed low investment

We report the event-study around the pre-programmed low investment first (round 12 pre-manipulation, round 13 post-manipulation): percentage return is modelled with relative time as a factor (t–2, t–1, t for the low investment, t+1, t+2) interacted with Condition and Game, controlling for the investment level. This separates the response at the low investment from changes before and after it.

#### Broader change across the post-manipulation game

To explore whether participants behaved differently in the RTG after the intervention compared to the control group, we model the percentage return (percentage of tripled investment returned to investor) using a linear mixed-effects model as described below:


$$\begin{array}{cc} {\text{Return}}_{\text{ij}}= & \beta_{0}+\beta_{1}{\text{ (Condition)}}_{i}+\beta_{2}{\text{ (Game)}}_{i}+\beta_{3}{\text{ (Investment)}}_{i}+\\ & \beta_{4}{\text{ (Condition × Game)}}_{i}+\beta_{5}{\text{ (Condition × Investment)}}_{i}+\beta_{6}{\text{ (Game × Investment)}}_{i}+\\ & \beta_{7}{\text{ (Condition × Game × Investment)}}_{i}+\beta_{8}{\text{ (Round)}}_{i}+\beta_{9}{\text{ (LowInvestment)}}_{i}+\\ & b_{0j}+b_{1j}{\text{ (Game)}}_{i}+b_{2j}{\text{ (Investment)}}_{i}+\epsilon_{\text{ij}} \end{array}$$

In R notation the model is Return ~ Condition * Game * Investment + Round + LowInvestment + (1 + Game + Investment | participant).

Models were estimated with afex (Singmann et al., 2022) using Kenward-Roger degrees of freedom; random-effects structures followed Matuschek et al. (2017). Post-hoc pairwise comparisons used Tukey HSD. We z-transform Investment so that the lower-order effects are easier to read in the presence of interactions. We call the slope of % return on the investment received the reciprocity slope. It measures how strongly a participant’s return depends on what the investor sent. A steep slope means strongly conditional cooperation, returning more only when the investor invests more. A flat slope means more unconditional cooperation. The Investment × Condition and Investment × Condition × Game terms test whether the intervention changed this slope.

The percentage return lies between 0 and 1 and is left-skewed, with about 6% of trials at zero. We use the Gaussian linear mixed model as the main analysis because it is straightforward to interpret and is standard in the trust-game literature. As a check, we refit it as a binomial mixed model, treating the return as a count out of the tripled endowment. The key Condition × Game and Condition × Game × Investment interactions hold in the same direction and remain significant (Supplement, Section 9.2).

#### Level of the effect: emotional reactivity, social appraisal, and strategy

For emotion self-reports, we analysed valence and arousal using linear mixed-effects models with fixed effects for Game (pre vs. post), Investment (z-scored), and their interaction, and participant-wise random intercepts and random slopes for Game. We also test whether the intervention changed how participants appraised the partner, using mixed-effects models of the four post-game investor ratings (cooperative, trustworthy, selfish, friendly) with Game and Condition as fixed effects and participant-wise random intercepts. Together with the reciprocity slope defined in Step 2, these separate whether the intervention acted on emotion, on appraisal, or on strategy.

#### Transfer to the Prisoner’s Dilemma

To test whether the effect transferred to a structurally different game, we predicted the probability of a cooperative action in the Repeated Prisoner’s Dilemma with a mixed-effects logistic regression, with Condition and pre- versus post-defection (rounds before or after the programmed defection) as fixed effects and a participant-wise random intercept.

#### Latent states and heterogeneity

Reciprocity unfolds over time and may reflect unobserved shifts in strategy, so we used hidden Markov models (Visser & Speekenbrink, 2022), fitted with the depmixS4 package (Visser & Speekenbrink, 2021), to recover latent behavioural states from each participant’s sequence of returns rather than imposing fixed categories. Each state s is a return policy: the proportion returned is modelled as a state-specific Gaussian with mean $\mu_{s}$ and standard deviation $\sigma_{s}$ , discretised over the proportions feasible given the current investment. The probability of a feasible proportional return r is the Normal mass between cut-points $r^{–}$ and $r^{+}$ set halfway to the neighbouring feasible proportions, normalised over the feasible range,


$$P\left(R_{t}=r|S_{t}=s\right)=\frac{\Phi \left(r^{+}|\mu_{s},\sigma_{s}\right)-\Phi \left(r^{-}|\mu_{s},\sigma_{s}\right)}{\Phi \left(r_{\max}^{+}|\mu_{s},\sigma_{s}\right)-\Phi \left(r_{\min}^{-}|\mu_{s},\sigma_{s}\right)},$$

with Φ the cumulative Normal. Transitions between states follow a multinomial-logistic function of the investment received $x_{t}$ and a contrast code $d_{t}$ whose value depends on the participant’s Condition-Game cell, defined per nested model below,


$$P\left(S_{t+1}=s^{\prime }|S_{t}=s,x_{t},d_{t}\right)=\frac{\exp\left(\beta_{0,s,s^{\prime }}+\beta_{1,s,s^{\prime }}x_{t}+\beta_{2,s,s^{\prime }}d_{t}\right)}{\sum_{s^{\prime \prime }}\exp\left(\beta_{0,s,s^{\prime \prime }}+\beta_{1,s,s^{\prime \prime }}x_{t}+\beta_{2,s,s^{\prime \prime }}d_{t}\right)},$$

so a model can test whether the intervention changed how investments move participants between states. We fitted five nested models that differ in where the transition dynamics may vary: HMM-full (all four Game × Condition combinations differ), HMM-coax (post-intervention versus the other three), HMM-ctrl (post-control versus the other three), HMM-prepost (first versus second game), and HMM-inv (investment only, with no Condition or Game differences). Concretely, $d_{t}$ is a contrast that flags the cells a model lets differ: in HMM-coax it equals 1 on post-intervention trials and 0 otherwise, so $\beta_{2,s,s^{\prime }}$ measures how much the intervention changes the probability of moving from state s to state $s^{\prime }$ . HMM-ctrl, HMM-prepost, and HMM-full use the corresponding contrasts, and HMM-inv omits $d_{t}$ . Pre-manipulation parameters are shared across conditions while post-manipulation parameters may differ by condition, so comparing these models tests whether the intervention changed the behavioural dynamics. We estimated 2 to 7 states for HMM-full by maximum likelihood via the EM algorithm and selected the number with the Bayesian Information Criterion (Schwarz, 1978), treating this as a fit-versus-complexity choice; it favoured 6 states. State labels were assigned after estimation, with states ordered by mean return. The Supplement (Section 11) gives the extended derivation and the per-model contrast coding.

## Behavioural results

We report the behavioural results in the order set out in the Methods. We first test whether the intervention changed the response to the withdrawal of trust, then how far that change extends across the game, then the background factors and the questionnaire moderators.

**Figure 2:**
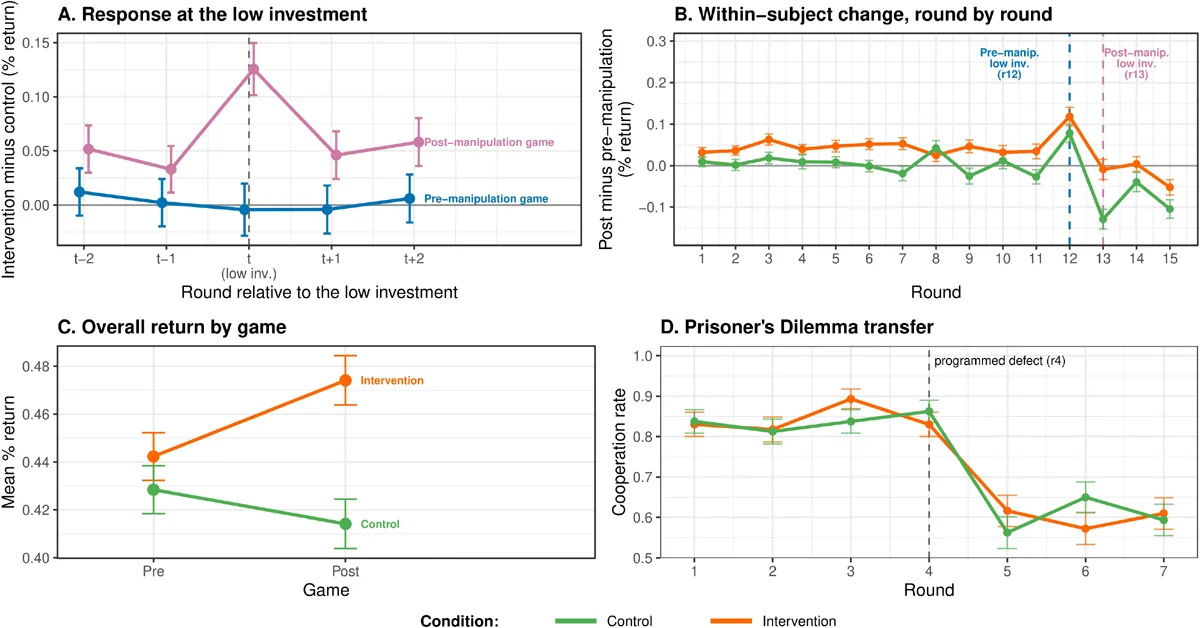
The behavioural effect, led by the response at the pre-programmed low investment. Colours: Game pre-manipulation blue, post-manipulation pink (Panel A); Condition Control green, Intervention orange (Panels B-D). Bands and error bars are plus or minus one standard error. **Panel A:** main behavioural test, the Intervention minus Control difference in percentage return around the pre-programmed low investment (time relative to that trial), for the pre- and post-manipulation games. **Panel B:** within-subject post minus pre percentage return by round and condition, with the round 12 and round 13 low investments marked. **Panel C:** estimated marginal mean percentage return by condition and Game (the Condition by Game interaction). **Panel D:** Repeated Prisoner’s Dilemma cooperation by round for both conditions, with the programmed defection at round 4 marked. The omnibus Condition by Game interaction reported in Panel C is the confirmatory (powered) test; Panel A shows where that effect is sharpest, at the low-investment trial.

After the intervention, the two conditions diverged in their response to the pre-programmed low investment (Figure 2A). That low investment was a genuine violation: fixed at 2 units, about 82% below the preceding investment of 11.4, and sent while the simulated investor was usually in a medium- or high-trust latent state (in a low-trust state on only 21% of these rounds), so it went against the agent’s own disposition. Returns tracked the absolute size of the investment received, while its deviation from recent rounds added no predictive value (Supplement, Section 9.3). On the low-investment round, the intervention group returned more than controls ( ΔM = 0.13, z = 5.21, p < .001), and the difference persisted one and two rounds later ( p = .036 and p = .008). Before the intervention the conditions did not differ at any time point around the low investment (all ps > .58). The pre-to-post change on the low-investment round was opposite in sign: the intervention group raised its return ( ΔM = 0.09, p < .001) while controls lowered theirs ( ΔM = –0.04, p = .015), and the Condition × Game interaction was clearly present in this window ( F (1, 1547.5) = 21.03, p < .001). The omnibus interaction for which the study was powered is reported below. Control trustees retaliated when the investor pulled back; the intervention group did not.

At the individual level, the same shift is visible round by round (Figure 2B), where the post-minus-pre change in return diverges between the conditions around the low investment. We define a retaliator as a participant whose mean return at and after the low investment fell below 30%. By this measure, the proportion of retaliators in the control group rose from 20.8% before the manipulation to 36.5% after it (McNemar $\chi^{2}$ (1) = 14.05, p < .001), whereas the intervention group changed little (17% to 20.1%, p = .51). The rise in retaliation after the manipulation was specific to the control group. This did not depend on the 30% cut-off: the control-group increase held at retaliator thresholds of 25%, 30%, and 35% (McNemar p < .001 at each), whereas the intervention group did not change at any of these thresholds (all p > .29) (Supplement, Section 9.4).

The effect was not confined to the low investment but was general across the post-manipulation game. The intervention group returned more overall (main effect of Condition: F(1,316.08)=8.41 , p=.004 ), and the difference was larger after the manipulation than before it (Condition × Game: F(1,316.08)=23.40 , p<.001 ). From the pre- to the post-manipulation game the intervention group increased its return ( ΔM=0.03 , 95% CI [0.02,0.05] , t(313.85)=4.64 , p<.001 ), whereas the control group decreased it ( ΔM=−0.01 , 95% CI [−0.03,0.00] , t(319.32)=−2.07 , p=.039 ). The groups matched before the manipulation ( t(316.29)=1.02 , p=.307 ), with investments and returns within the range reported for trust games (investments around 40% to 60% of the endowment, returns around 35% to 50% of the amount received; Cochard et al. (2004); Charness et al. (2008)), and differed after it ( t(316.05)=4.26 , p<.001 ; Figure 2C).

In both groups, returns rose with the size of the investment received (main effect of Investment: F(1,337.35)=41.05 , p<.001 ), declined over successive rounds (main effect of Round: F(1,8632.78)=106.01 , p<.001 ), and were lower on the pre-programmed low investment rounds themselves ( F(1,8628.87)=21.82 , p<.001 ; ΔM=0.03 , 95% CI [0.02,0.04] , z=4.67 , p<.001 ).

The questionnaire moderation results were mostly null, but not entirely. Adding borderline personality traits (PAI-BOR), emotion-regulation difficulties (DERS), and reflective functioning (RFQ-8), with their interactions with Condition, Game, and Investment, left the Condition × Game effect essentially unchanged (Supplement, Section 5). PAI-BOR and DERS did not show significant trait interactions in this return model. The RFQ-8 model contained two significant secondary interactions involving RFQ-8 (RFQ-8 × Game × Condition and RFQ-8 × Condition × Investment), but the four-way RFQ-8 × Game × Condition × Investment term was not significant. Because these trait models were exploratory and not corrected for multiple testing, we treat the RFQ-8 pattern as hypothesis-generating rather than evidence that reflective functioning reliably moderated the intervention effect. In the targeted analyses (Supplement, Section 5.2), the link between perceived investor selfishness and return was robust across the sample ( F (1, 513.4) = 15.54, p < .001) and was not moderated by any of the three trait measures, and questionnaire scores did not predict who adopted a retaliatory strategy after the pre-programmed low investment. At the trial level of emotion, however, the relation between reported valence or arousal and the investment received was moderated by PAI-BOR and DERS in a game-dependent way. Only the intervention group rated emotion each round, so we have no control comparison for this pattern; we treat it as exploratory and not as a mechanism behind the intervention effect (Supplement, Section 5.2).

## Mechanism

**Figure 3:**
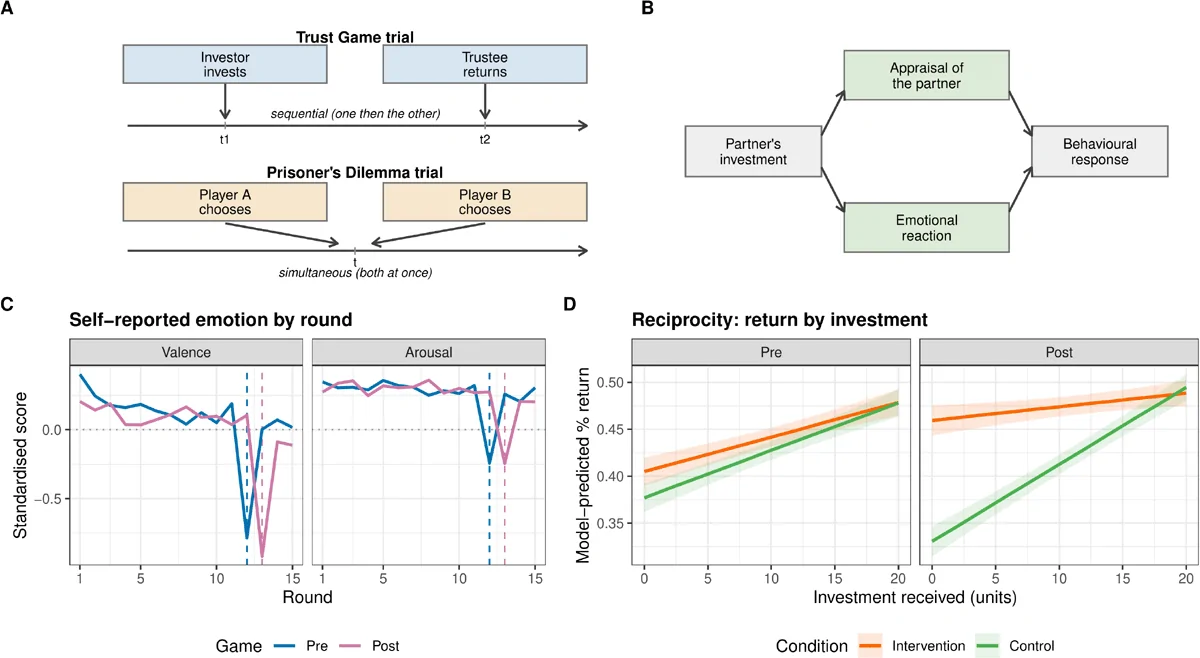
From task structure to mechanism. **Panel A:** the decision structures of the two games on a common time axis; in a Trust Game trial the investor acts at time t1 and the trustee responds at t2 (sequential), whereas in a Repeated Prisoner’s Dilemma trial both players choose at a single time t (simultaneous), so the absence of transfer between the games is expected rather than a failure of the intervention. **Panel B:** a conceptual model of how the trustee may reason through the game, from the partner’s investment, through a latent appraisal of the partner (a belief about the investor) and an emotional reaction, to the behavioural response; this is a hypothesised process, not a measured mediation (emotion is measured each round in the intervention group; the latent appraisal is probed only through the investor ratings collected once after each game). **Panel C:** self-reported emotion (valence and arousal, z-scores) in the intervention group by round, pre- and post-manipulation, with the low investments at rounds 12 and 13 marked; emotion did not change with the intervention. **Panel D:** model-predicted percentage return as a function of the investment received, by condition and game; after the manipulation the control group’s return depends more steeply on the investment received while the intervention group’s is flatter, that is, less conditional reciprocity. Colours: in Panel C, game pre-manipulation blue and post-manipulation pink; in Panel D, Control green and Intervention orange. Bands and error bars are plus or minus one standard error.

We next asked at what level the intervention acted, following the process sketch in Figure 3B. It was not in emotional reactivity. In the intervention group, who rated their emotion on each round, higher investments brought more positive ( F(1,3448.17)=2108.08 , p<.001 ) and more aroused ( F(1,3453.24)=1505.03 , p<.001 ) emotion, and both valence ( F(1,117.20)=17.99 , p<.001 ) and arousal ( F(1,117.19)=5.52 , p=.021 ) declined from the pre- to the post-manipulation game. Neither the valence ( F(1,3419.70)=1.49 , p=.222 ) nor the arousal ( F(1,3409.69)=0.12 , p=.726 ) response to investment shifted with the manipulation, so the emotional response to what the investor sent was unchanged while percentage returns increased (Figure 3C). This null interaction held when within-game round was added as a covariate (valence F(1,3422.41)=0.81 , p=.367 ; arousal F(1,3410.09)=0.03 , p=.864 ).

The intervention also did not change how participants saw the investor. In the second game all participants rated the investor as less cooperative ( ΔM=−0.42 , 95% CI [−0.69,−0.15] , t(317)=−3.10 , p=.002 ), less trustworthy ( ΔM=−0.43 , 95% CI [−0.70,−0.16] , t(317)=−3.19 , p=.002 ), less friendly ( ΔM=−0.40 , 95% CI [−0.64,−0.17] , t(317)=−3.36 , p<.001 ), and more selfish ( ΔM=0.36 , 95% CI [0.10,0.61] , t(317)=2.76 , p=.006 ). The intervention group rated the investor more cooperative ( ΔM=0.44 , 95% CI [0.04,0.84] , t(317)=2.16 , p=.031 ) and less selfish ( ΔM=−0.41 , 95% CI [−0.80,−0.02] , t(317)=−2.04 , p=.042 ) overall, but no rating showed a Condition × Game interaction (all ps > .22). As such, the intervention effect was unlikely to be due to a change in how the partner was seen.

What changed was the strategy itself. The control group reciprocated investments more steeply than the intervention group (Investment × Condition F(1,315.26)=8.27 , p=.004 ; ΔM=−0.02 , 95% CI [−0.04,−0.01] , z=−2.88 , p=.004 ), and only after the manipulation (three-way Game × Condition × Investment F(1,8855.73)=24.89 , p<.001 ). The groups reciprocated alike before the manipulation ( ΔM=−0.01 , 95% CI [−0.02,0.01] , z=−0.90 , p=.366 ); after it the intervention group’s return depended less on the investment and the control group’s more ( ΔM=−0.04 , 95% CI [−0.06,−0.02] , z=−4.47 , p<.001 ), a reliable slope change ( ΔM=0.03 , 95% CI [0.02,0.04] , z=4.99 , p<.001 ). Cooperation in the intervention group thus became less dependent on what the investor sent (Figure 3D).

This reciprocity result rests on the investment received, which is not exogenous, because the simulated investor adapts to what it receives back. The investor sent more to the intervention group ( F(1,316)=8.88 , p=.003 ) and more in the post-manipulation game ( F(1,316)=7.80 , p=.006 ), following from their higher returns. To keep this from confounding the comparison, the investment received enters every return model as a covariate, so the intervention-versus-control difference is estimated at a matched level of investment rather than reflecting the higher investments the intervention group drew (Supplement, Section 7).

When asked during debrief whether they thought the investors they faced were human or not, 44% of participants thought they were either facing a human (22%) or were not sure of the nature of the co-player (22%). Many answers reflected participants projecting human traits such as “spitefulness” or “greed” onto the artificial co-player’s behaviour.

To test whether the intervention effect depended on participants’ beliefs about facing a human versus computerised opponent, we ran a moderation analysis including belief category (believed machine, believed human, or unsure) as an additional factor (Supplement, Section 10). Neither the three-way interaction (Condition × Game × Belief; *F*(2, 313.3) = 0.5, *p* = .61) nor the four-way interaction with Investment (*F*(2, 8873.5) = 0.26, *p* = .77) was significant, indicating that the intervention effect did not depend on whether participants believed they were facing a human or machine. In the believed-human subgroup the between-group difference at post-manipulation was in the same direction but did not reach conventional significance ( ΔM = 0.051, *p* = .09), and within-condition the intervention group increased its returns (*p* = .007) while the control group showed a smaller, non-significant change in the opposite direction (*p* = .11), the smaller subgroup limiting power for the control comparison. The pattern is consistent with the same effect operating across belief subgroups, with reduced power in the smaller ones. The intervention effect therefore does not appear to depend on participants’ awareness of computerised opponents.

## Transfer to the Repeated Prisoner’s Dilemma game

In the Repeated Prisoner’s Dilemma, we modelled cooperative choice with a mixed-effects logistic regression, using Condition and pre- versus post-defection (before or after the defection trial) as fixed effects and a participant random intercept. Cooperation declined after the partner’s defection, $\chi^{2}\left(1\right)=237.67$ , p<.001 , but there was no main effect of Condition, $\chi^{2}\left(1\right)=0.10$ , p=.754 , and no Condition × defection interaction, $\chi^{2}\left(1\right)=0.23$ , p=.635 (Figure 2D). The task is structurally different from the Trust Game because choices are simultaneous rather than sequential (Figure 3A). The RPD had only 7 rounds and was not independently powered for transfer, so this null should be interpreted cautiously.

## HMM analysis of participant returns

**Figure 4:**
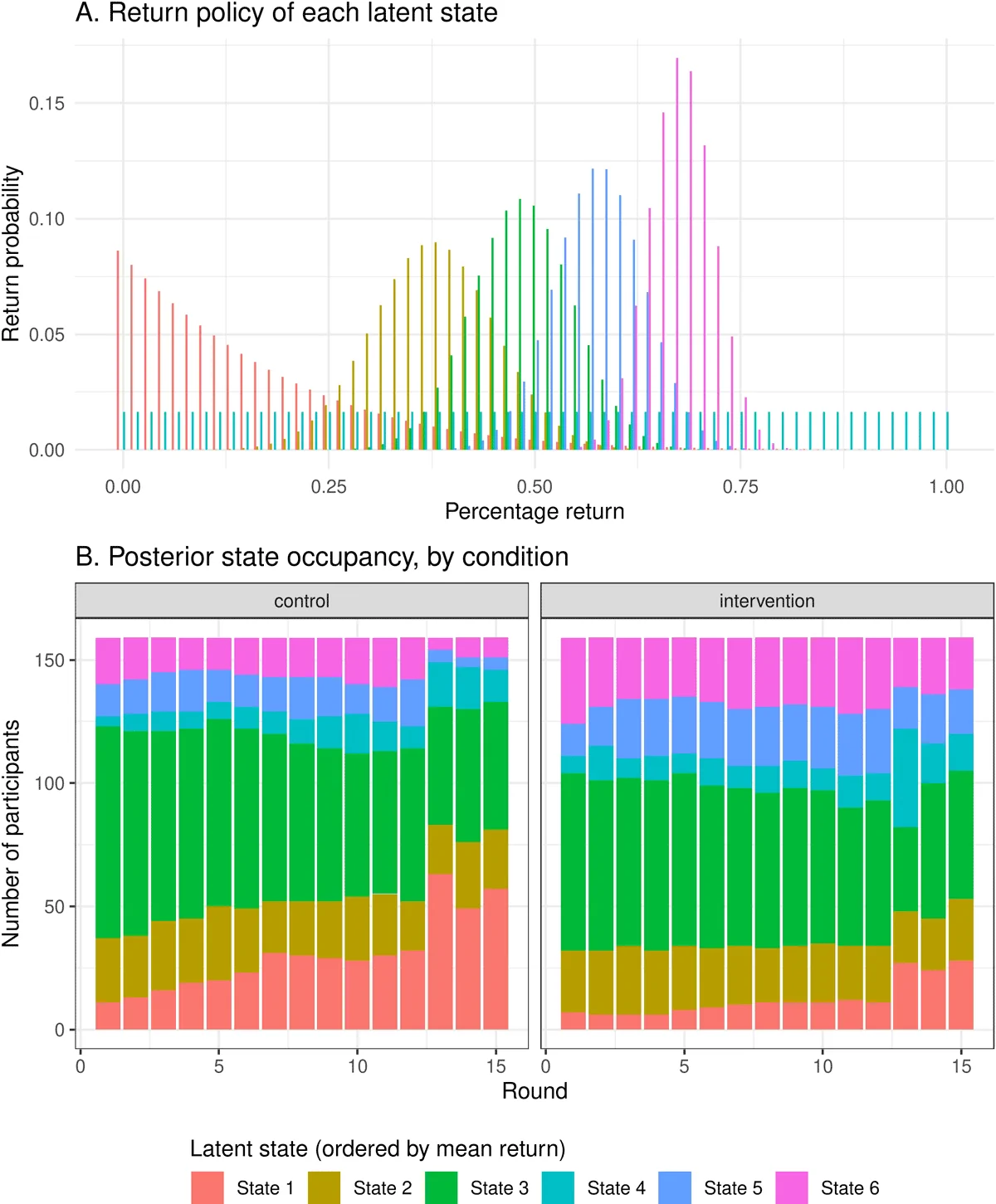
**Panel A:** the distribution of participants’ percentage return for each of the latent states in the 6-state HMM-full model shows distinct policies centred around different return levels. The latent states are ordered by the mean of the discretised Gaussian representing the policy in that state, so higher numbered states can be considered more pro-social. **Panel B:** This figure characterises behavioural differences between conditions as the result of participants being in more pro-social (higher return) states in the intervention condition compared to the control condition both before and after the low investment trial. The distribution of posterior trustee states post-manipulation by condition for all rounds, as estimated by the most likely posterior state in the best fitting HMM model (HMM-full) using a local decoding procedure is visibly more weighted towards higher return states. As in Panel A, states here are represented in increasing degrees of average percentage returns from the lowest (state 1) to the highest (state 6) return state.

The participant HMM mirrors the conceptual structure of the investor HMM in Figure 1B and 1C: discrete latent states with distinct return policies, and transitions between them driven by the investment received. The states here are participant-side return policies (Figure 4A) rather than investor-side investment policies. To assess how the intervention changed participants’ state dynamics, we compared the five hidden Markov models specified in Methods (Latent states and heterogeneity).

Focusing on models with 6 latent states, likelihood ratio tests showed that the HMM-full model, in which transition dynamics are free to differ across all four Game × Condition cells, fitted significantly better than four more constrained alternatives. It improved on an investment-only baseline with no Condition or Game differences (HMM-inv, $\chi^{2}\left(120\right)=231.05$ , p < .001); on a model in which only the control group’s post-manipulation transitions differ from the rest (HMM-ctrl, $\chi^{2}\left(60\right)=108.44$ , p<.001 ); on one in which only the intervention group’s post-manipulation transitions differ (HMM-coax, $\chi^{2}\left(60\right)=129.85$ , p<.001 ); and on one in which transitions differ between the first and second game but not between conditions (HMM-prepost, $\chi^{2}\left(60\right)=110.01$ , p<.001 ). As such, there is evidence that participants reacted differently to the investments in all four combinations of Condition and Game. Consistent with the mixed-effects trends, the intervention was associated with reduced sensitivity to investment levels and a shift in posterior state occupancy toward higher-return states (see Figures 3D and 4). The estimated state-dependent policy of trustee actions, according to the HMM-full model, is depicted in Figure 4A.

The HMM analysis of participant behaviour reveals additional insights into the previous aggregate analyses: specifically, it shows individual-level heterogeneity in behavioural states and how participants transition between cooperative and non-cooperative patterns across rounds. Taking the best-fitting 6-state HMM-full model, we used a local decoding procedure to determine each participant’s most likely state on each round of the RTG. The states are ordered by expected return, with state 1 having the lowest mean return and state 6 the highest. While the mixed-effects analyses above show that mean returns differ between conditions, the HMM analysis reveals *why*: participants in the intervention condition were less likely to become “stuck” in low-return behavioural states following the pre-programmed low investment, the precise pattern the intervention targeted. Figure 4B shows that participants were more likely to be in a lower return state in the control condition compared to the intervention condition, both before and after the low investment trial. For instance, in round 5, state 1 was the most likely state for only 5% of participants in the intervention condition compared to 13% in the control condition ( $\chi^{2}\left(1\right)=$ 4.74, *p* = .029). For the trial after the post-manipulation low investment (round 14), state 1 was the most likely state for only 15% of participants in the intervention condition compared to 31% in the control condition ( $\chi^{2}\left(1\right)=$ 10.24, *p* = .001). While the posterior states indicate that the intervention was effective, a non-negligible proportion of participants in the intervention condition did not exhibit the coaxing behaviour promoted by the intervention. Directly following the low investment in round 13, 17% of participants in the intervention condition were assigned to state 1 with the lowest average returns, highlighting individual differences in the effectiveness of the intervention. This heterogeneity did not track the trait measures. Occupying the lowest-return state on the trial after the post-manipulation low investment was not predicted by borderline traits (PAI-BOR OR = 1.05, p = .73), emotion-regulation difficulties (DERS OR = 1.08, p = .56), or reflective functioning (RFQ-8 OR = 0.95, p = .70), each adjusted for Condition. The intervention itself, by contrast, reduced the odds of being stuck there ( OR = 0.40, p = .001). It was the manipulation, not pre-existing traits, that governed who remained in the most retaliatory state (Supplement, Section 5.2).

We checked how well the model reproduces the observed behaviour. Simulating returns from the fitted model reproduced the observed mean returns by round and by Condition × Game (mean absolute error 0.014, with 51 of 60 cells inside the 95% predictive interval), and a recovery analysis showed that the state return policies and decoded state occupancy are recovered well, while the precise emission and transition parameters are recovered only weakly. We therefore order states by mean return and read the latent states descriptively, in terms of their return policies and occupancy, rather than as precise parameter estimates. The Supplement (Section 11.2) reports the full check and explains why the raw parameters are only weakly identified.

## Discussion

This proof-of-concept study shows that a brief DBT-inspired cognitive intervention can enhance cooperative behaviour in the RTG. Following the intervention, participants increased their returns without a corresponding change in emotional response, indicating the intervention’s effectiveness in preserving cooperation and reducing retaliation to a breach of cooperation. Those in the control condition returned similar proportions before the programmed low investment but reduced their returns afterward. Returns rose by about 6 percentage points relative to controls (from 41.4% to 47.4%, a 14.5% relative increase), against typical RTG baselines of 35 to 50 percent (Johnson & Mislin, 2011). The effect exceeds the 4-point gain from limited (numerical-only) communication but is smaller than the 11-point gain from full (chat-plus-numerical) communication reported in trust games (Ben-Ner et al., 2011). Beyond the magnitude effects, an HMM analysis showed that those in the intervention condition were less prone to low-return states following the pre-programmed low investment. This aligns with the intervention’s target: participants were less likely to get stuck in the low-return states that typically follow a breach of cooperation.

The intervention changed how trustees respond after a low investment. A brief, explicit, DBT-inspired prompt not to retaliate produced a general increase in returns across the post-manipulation game, with the two groups diverging in opposite directions at the pre-programmed low investment. Most simply, affect and action dissociated: the intervention reduced retaliation after the investor’s apparent withdrawal of trust without a corresponding change in self-reported negative emotional reactivity or partner appraisal. The negative reaction to the low investment was preserved; what changed was whether it was expressed as retaliation, the signature of DBT’s opposite-action principle.

The increase is unlikely to reflect general learning, since returns by control participants did not rise. Both groups faced the same investor algorithm; the investor reacts to higher returns but follows the same policy, and changes in post-game investor ratings did not differ between conditions, so differential investor behaviour and differential partner appraisal are unlikely to account for the effect. The subgroup analysis further showed the effect did not depend on whether participants believed they were facing a human or machine, so it is not an artefact of computerised opponents.

In the intervention group, who rated emotion each round, we observed a decrease in both emotional positivity and arousal in the second RTG compared to the first. This general decline in emotional intensity is likely attributable to factors such as decreased engagement or increased fatigue as the experiment progressed, rather than being a specific effect of the intervention. Despite this reduction in emotional intensity, intervention participants maintained higher levels of cooperative behaviour, and their emotional reactivity to the investment received did not shift with the manipulation. The investor ratings showed no Condition by Game interaction either, so neither the emotional response to the investment nor the appraisal of the partner moved with the manipulation.

Because the prompt is explicit, we cannot exclude that part of the effect reflects following a clear instruction, with no latent therapeutic mechanism engaged; the contribution is the behavioural specificity of the change. An instructional component would not on its own make that change uninteresting: directive instruction and the expectations it sets are active ingredients of evidence-based psychotherapy (Beck, 2011; Trachsel & Grosse Holtforth, 2019), and even an explicit prompt is taken up unevenly, as the heterogeneous response here shows. The intervention group returned more across the post-manipulation game; at the pre-programmed low investment the two groups moved in opposite directions, the control group showing stronger retaliation while the intervention group upheld its returns; the change did not transfer to the Repeated Prisoner’s Dilemma; and it occurred without a measurable change in self-reported emotional reactivity. Most likely, this reflects a reappraisal of the strategic value of not retaliating: the emotional reaction was preserved while the retaliatory response fell, a change in how feeling translates into action rather than a covert DBT mechanism.

The lack of transfer to the Prisoner’s Dilemma warrants careful interpretation. Three readings are possible. First, the two tasks may engage partly different constructs. As trustees, participants respond to a partner’s extended and then withdrawn trust, a reactive, repair-oriented form of reciprocity; the Repeated Prisoner’s Dilemma instead requires a proactive, simultaneous strategic choice (Figure 3A) (Doppelhofer et al., 2025). On this reading the absence of transfer may be expected, because the prompt targets the response to a low investment, which the Repeated Prisoner’s Dilemma does not pose in the same way. Second, the two games differ in strategic structure. In the Repeated Trust Game the adaptive investor rewards generous returns with higher future investments, a graded and immediate contingency. The brief, binary Prisoner’s Dilemma against a tit-for-tat partner offers a much coarser and sparser version of that incentive, so the lesson of not retaliating is harder to apply there. Third, because the intervention prompt itself referred to the Trust Game, this lack of transfer is consistent with a task-specific instruction effect rather than a generalised therapeutic skill, and the present design cannot distinguish that account from the two above.

Analysing participants’ behaviour with hidden Markov models, we found clear individual differences in how returns changed between the pre- and post-manipulation RTG, which can be seen as a proxy for the effectiveness of the intervention. Some participants may not have been convinced that coaxing via high returns was a good way to establish cooperation and decided to reduce their returns in the second trust game. Their impulse to “punish” the other player for a trust withdrawal may have been too strong to be overridden by the intervention. This was also evident in participants’ post-intervention replies about whether they would change their behaviour: roughly a third declared a shift toward cooperation, and declared change predicted the actual pre- to post-manipulation change in low-investment-window returns (replies coded by Claude Opus 4.7 in two blind passes with co-author adjudication; see Methods and Supplement, Section 9.6). Heterogeneity in response to treatment is common in psychiatry and related fields. Such heterogeneity may reflect the multi-determined nature of mental-health processes, which the network-theory perspective treats as interacting neural, cognitive, and contextual factors (Fried & Cramer, 2017). This view was used to justify computational psychiatry’s difficulty in establishing differential and reliable predictors of treatment response (Hitchcock et al., 2022). Here, we found heterogeneity in reaction to a relatively explicit intervention by a sample of participants from the general population. This suggests that the issue of variable response to treatment may result from the interaction of two sources of variability: the phenotyping of the disorder as well as the phenomenological aspects of the intervention itself. As such, a rigorous exploration of the determinants of inter-individual differences in response to an intervention in the general population is required.

These individual differences notwithstanding, our findings have broader implications for understanding therapeutic mechanisms. They show that a brief, explicit prompt built on a Dialectical Behaviour Therapy principle, acting against the urge to retaliate, can change reactions to a breach of cooperation in a highly controlled online experimental setting. These effects emerged in individuals without clinical diagnoses. The clinical literature indicates DBT effectively treats borderline personality disorder, a condition characterised by profound deficits in repairing social trust after interpersonal violations (King-Casas et al., 2008). Our results are consistent with the rationale of this therapeutic approach.

Several recent studies have used computational and experimental methods to investigate psychotherapeutic mechanisms: cognitive interventions influence effort sensitivity (Norbury et al., 2024), cognitive distancing shapes computational learning mechanisms (Dercon et al., 2024), and behavioural activation relates to Pavlovian biases (Huys et al., 2022). The intervention’s task-specificity (Trust Game but not Prisoner’s Dilemma) suggests that future work could target specific social contexts.

Our methodological approach using HMM-based computerised agents offers advantages that complement the need for eventual human-human replication. First, the HMM agents were trained on actual human investor data, ensuring behaviourally realistic responses while maintaining complete experimental control, a combination that is not possible with human partners. Second, the pre-programmed low investment on specific rounds allowed us to precisely examine responses across conditions with identical timing and magnitude. Third, this approach enabled a fully online study with large sample sizes at relatively low cost, establishing the intervention’s feasibility before investing in more resource-intensive human dyad studies. This agent class has also supported individual-difference studies of strategic exploitation in extended-horizon trust interactions (Guennouni et al., 2026). While replication with human partners remains an important next step to establish ecological validity, this proof-of-concept study shows that the intervention has a measurable effect under controlled conditions.

Several limitations should be noted. Our sample comprised non-clinical adults recruited online from predominantly Western countries, and generalisability to non-WEIRD populations or clinical groups (e.g., those with borderline personality disorder) remains to be established. The intervention is a single explicit prompt combining psychoeducation, reflection on consequences, and behavioural guidance. The design cannot separate a DBT-specific skill from the effect of clear instruction, nor isolate which element drove the change; a study that varied its components would be needed for that. The result therefore shows that an explicit anti-retaliation prompt changes behaviour in this paradigm; it does not show that a DBT-specific mechanism has been isolated. Whether a comparable prompt helps people who struggle to repair cooperation in clinical or everyday interpersonal contexts, for example in borderline personality disorder, is an open question for studies in those populations (De Panfilis et al., 2025). Although the computerised investor was not a human partner, results may not generalise to interactions with real people, who bring theory of mind, strategic sophistication, and emotional reactivity. A further asymmetry is that intervention participants completed an emotion evaluation grid on every round, while control participants evaluated the investment on different dimensions. This continuous self-monitoring could contribute to the observed effects beyond the discrete intervention content. The control rating, however, veridically tracked the objective investment received, so its upward trajectory across rounds reflects the investor sending more after higher returns rather than a rating-induced drift (Supplement, Section 9.5). Finally, data collection occurred entirely online, and laboratory settings with in-person interactions might yield different results.

Future studies could refine such cognitive interventions, possibly making them more interactive and engaging. Testing such interventions with participants who struggle to repair relationships after trust breakdowns, such as those with Borderline Personality Disorder, could be particularly valuable. The relative ease of delivering online interventions and repeated interactions and training with artificial but human-like agents opens up possibilities for efficient, low-cost treatment programmes to help a wide variety of people overcome tendencies for detrimental actions in social situations. More broadly, the HMM-agent paradigm demonstrated here offers a reusable experimental tool for researchers studying trust dynamics, intervention mechanisms, or social learning under conditions requiring both experimental control and behavioural realism.

## Additional File

The additional file for this article can be found as follows:

10.5334/cpsy.178.s1 Supplementary File 1. Supplementary Information containing additional methodological details, questionnaire analyses, robustness checks, subgroup analyses, and hidden Markov model validation.

## Data Availability

Analysis code, figures, and supplementary materials are available on OSF (https://osf.io/wfz52). The anonymised dataset is available upon request from the corresponding author.
