# Supplementary material for: (Re)building Cooperation: Effects of a Cognitive Intervention on Cooperative Behaviour in Games
Source: Comput Psychiatr. 2026 Sep 16;10(1):184–201. doi: 10.5334/cpsy.178 (PMC13588210; doi:10.5334/cpsy.178)
Supplement: Supplementary file 1 [file cpsy-10-1-178-s1.pdf]

(Re)building cooperation: Effects of a cognitive intervention on cooperative behaviour in games

Supplementary Information

1 Repeated Trust Game

Participants played a 15-round repeated trust game (Berg et al., 1995) in the trustee role against a computer-programmed investor. Each player was represented with an icon with the participant always on the left of the screen and the opponent on the right. The participants were able to choose the icon that represents them at the start of the experiment. The icon representing the opponent changed at the start of each new game, to simulate a new interaction partner. Participants were not told they were facing computerised opponents. We chose to simulate the behaviour of a human interaction partner through allowing for a delay while pairing with new opponents at the start of each game as well as programming the agents to respond during each round after a random time lapse (randomly chosen between 5 and 10 seconds).

On each round of the RTG, before deciding how much to return, participants were asked to provide feedback on the investment sent. This feedback consisted of a rating on two dimensions, provided via a two-dimensional field. What the axes of the field represented differed between the conditions. In the intervention condition, participants were asked to rate their emotional reaction to the other player's choice of investment in terms of valence (positive or negative) and arousal (low or high). In the control condition, participants were asked to rate the choice of investment in terms of speed (slow or fast) and magnitude (low or high). At the beginning of each game, participants were provided with detailed explanations of the meaning of the two axes as well as the opportunity to provide a baseline emotional state through using the field prior to the start of the game. Participants in the control condition were asked to choose any point on the two dimensional grid to show they have understood how to interact with the grid.

2 Repeated Prisoner's Dilemma

The other game the participants played is the normal form, double-choice Repeated Prisoner's Dilemma (RPD). Over multiple rounds, participants could choose one of two actions: A cooperative action that would yield a high pay-off if the other person also cooperated, and the lowest possible pay-off if they did not cooperate, or a non-cooperative option that would yield a high pay-off if the other person chooses the cooperative action and a lower pay-off if they also defect. Figure S1 shows the payoff of each combination of actions as presented to the participants. As an opponent in this game, we used an artificial agent that played according to Tit-For-Tat (Axelrod & Hamilton, 1981). It started by choosing the cooperative action and then it mirrored whatever the other player played in the previous round.

3 Emotion Grid for the Intervention Condition

Immediately before making return decisions in the RTG, participants are shown a two dimensional grid where they need to report their reaction to the investment. Those in the intervention condition were asked questions about their emotional state (Figure S2) and those in the control condition were asked to report features of the investment unrelated to their emotion.

		Their decision	
		A	B
Your decision	A	You receive: 2 points	You receive: 7 points
		They receive: 2 points	They receive: 1 points
	B	You receive: 1 points	You receive: 5 points
		They receive: 7 points	They receive: 5 points

Figure S1: Screenshot of the Repeated Prisoner's Dilemma game. Over multiple rounds, participants could choose one of two actions: A cooperative action that would yield a high pay-off if the other person also cooperated, and the lowest possible pay-off if they did not cooperate. Or a non-cooperative option that would yield a high pay-off if the other person chooses the cooperative action and a lower pay-off if they also defect. Shown here is the table explaining the payoffs of each combination of actions the participant and their opponent choose

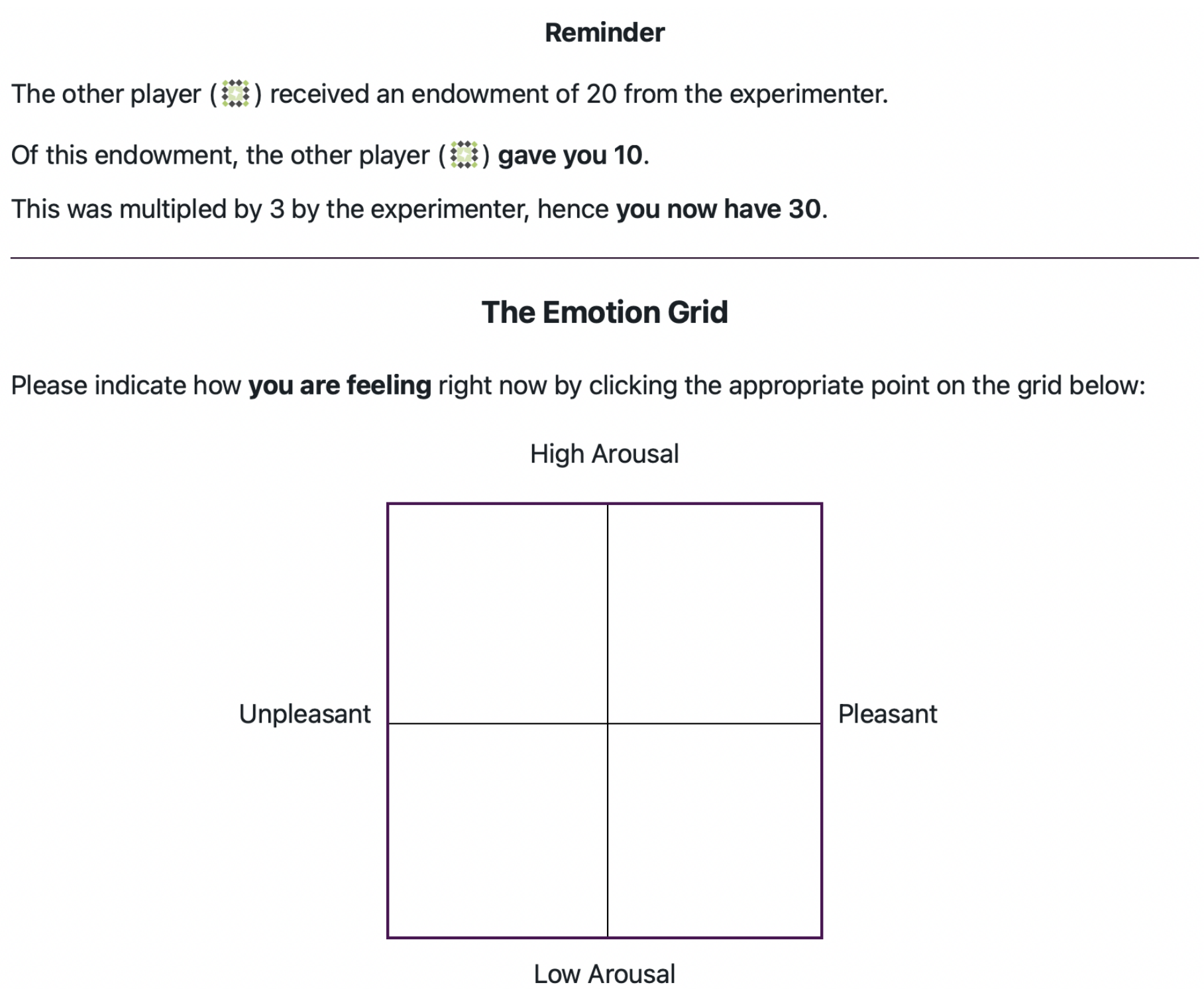

Figure S2: Screenshot of the emotion grid for the intervention condition

4 Conceptual schematic: trust-game versus Prisoner's Dilemma trial, and the mechanistic chain

The conceptual schematic that contrasts a single Trust Game trial with a single Repeated Prisoner's Dilemma trial, and summarises the mechanistic chain from the partner's behaviour through appraisal and emotion to the trustee's response, is presented in the main text (Figure 4), to which the reader is referred.

5 Self-report questionnaires

We had directional a-priori expectations for the individual-difference measures. Borderline personality features are associated with heightened sensitivity to social rejection and with altered appraisal of, and difficulty maintaining, interpersonal trust (Preti et al., 2023). In the trust game specifically, trustees with borderline features give lower returns and fail to repair cooperation after a breach (King-Casas et al., 2008). We therefore expected participants with higher borderline traits (PAI-BOR), greater emotion-regulation difficulties (DERS), or lower reflective functioning (RFQ-8) to retaliate more after the pre-programmed low investment, to show a larger drop in returns from the pre- to the post-manipulation game, to benefit less from the intervention, or to show greater emotional reactivity to the investment received. We test these expectations first as moderation of the behavioural effect, and then more specifically as moderation of emotional reactivity, of the perceived-selfishness to return link, and of post-low-investment retaliation.

We tested whether scores on the PAI-BOR, DERS, and RFQ8 moderated the intervention effect by including each Z-transformed questionnaire score as an additional fixed effect in the mixed-effects model of percentage returns. Each model included the questionnaire score, Condition (intervention or control), Game (pre or post manipulation), Investment (z-scored), and all interactions, with participant-wise random intercepts.

5.1 Moderation of the intervention effect by trait scores

The questionnaire moderation results were mostly null, but not entirely. Adding PAI-BOR, DERS, or RFQ-8 (with all interactions with Condition, Game, and Investment) to the mixed-effects return model left the Condition $\times$ Game effect essentially unchanged. In the PAI-BOR and DERS models, no interaction involving the trait score reached significance. In the RFQ-8 model, two secondary interactions reached significance: RFQ-8 $\times$ Game $\times$ Condition and RFQ-8 $\times$ Condition $\times$ Investment. The four-way RFQ-8 $\times$ Game $\times$ Condition $\times$ Investment term, which most directly tests whether RFQ-8 changes the intervention-related pre-to-post investment-slope pattern, was not significant. Because these models are exploratory and include many uncorrected interaction tests, we interpret the RFQ-8 terms as hypothesis-generating rather than confirmatory moderation evidence. Full ANOVAs are in Tables S1-S3.

5.2 Targeted moderation analyses: emotion-investment coupling, perceived selfishness, and post-low-investment retaliation

Three targeted analyses ask whether borderline traits (PAI-BOR), emotion-regulation difficulties (DERS), or reflective functioning (RFQ-8) moderate specific links in the trustee process, even though they do not moderate mean returns. These analyses are exploratory, non-preregistered, and not corrected for multiple testing; we report them as hypothesis-generating rather than confirmatory.

5.2.1 Trait modulation of trial-level emotion-investment coupling

We tested whether DERS (Difficulties in Emotion Regulation Scale; higher score means greater difficulty regulating emotion) or PAI-BOR (Personality Assessment Inventory, Borderline subscale; higher score means more borderline personality features) moderate the trial-level coupling between investment received and immediately-reported emotion. The models were fit on the intervention group only (the control group rated investment magnitude, not emotion), with a random investment slope per participant. Figure S3 shows the simple slopes for both traits and both emotion outcomes; the two trait-by-trait paragraphs below give the corresponding interaction tests.

Table S1: ANOVA results for the mixed-effects model of percentage return with PAI-BOR moderation

Effect	df1	df2	F	p
PAI-BOR	1	315.3	0.72	.398
Game	1	9070.8	6.84	.009
Condition	1	315.2	9.37	.002
Investment	1	9209.8	427.18	< .001
PAI-BOR × Game	1	9071.3	0.19	.663
PAI-BOR × Condition	1	315.3	0.47	.495
Game × Condition	1	9070.8	66.44	< .001
PAI-BOR × Investment	1	9204.8	1.80	.180
Game × Investment	1	9161.6	4.70	.030
Condition × Investment	1	9209.8	35.47	< .001
PAI-BOR × Game × Condition	1	9071.3	0.02	.880
PAI-BOR × Game × Investment	1	9160.6	1.16	.282
PAI-BOR × Condition × Investment	1	9204.8	0.02	.889
Game × Condition × Investment	1	9161.6	24.01	< .001
PAI-BOR × Game × Condition × Investment	1	9160.6	0.78	.376

Table S2: ANOVA results for the mixed-effects model of percentage return with DERS moderation

Effect	df1	df2	F	p
DERS	1	315.3	0.01	.917
Game	1	9071.1	6.82	.009
Condition	1	315.2	9.51	.002
Investment	1	9208.3	424.17	< .001
DERS × Game	1	9071.5	0.29	.588
DERS × Condition	1	315.3	0.02	.880
Game × Condition	1	9071.1	64.95	< .001
DERS × Investment	1	9206.8	3.41	.065
Game × Investment	1	9161.6	4.82	.028
Condition × Investment	1	9208.3	34.74	< .001
DERS × Game × Condition	1	9071.5	2.70	.100
DERS × Game × Investment	1	9165.1	0.47	.493
DERS × Condition × Investment	1	9206.8	0.06	.814
Game × Condition × Investment	1	9161.6	25.07	< .001
DERS × Game × Condition × Investment	1	9165.1	1.77	.183

DERS (greater emotion-regulation difficulty). Participants with higher DERS scores showed a stronger positive coupling between the investment received and immediately-reported valence in the post-manipulation game; the pre-manipulation game showed no such trait gradient (valence × DERS × Game $F(1, 4516.5) = 8.25$, $p = .004$). For arousal, the pattern was reversed in time: higher-DERS participants showed a weaker arousal-investment coupling in the pre-manipulation game, and this gradient attenuated after the manipulation (arousal × DERS × Game $F(1, 4502.0) = 15.72$, $p < .001$).

PAI-BOR (more borderline personality features). For valence, the PAI-BOR pattern paralleled DERS: in the post-manipulation game, higher-PAI-BOR participants showed a stronger valence-investment coupling; pre-manipulation slopes were uniform across the trait (valence × PAI-BOR × Game $F(1, 4514.9) = 4.27$, $p = .039$). For arousal, PAI-BOR moderated the slope but not in a game-dependent way: the 2-way PAI-BOR

Table S3: ANOVA results for the mixed-effects model of percentage return with RFQ-8 moderation

Effect	df1	df2	F	p
RFQ-8	1	315.2	1.22	.271
Game	1	9070.7	6.39	.012
Condition	1	315.1	9.16	.003
Investment	1	9208.4	436.97	< .001
RFQ-8 × Game	1	9071.0	0.18	.673
RFQ-8 × Condition	1	315.2	0.72	.396
Game × Condition	1	9070.7	66.43	< .001
RFQ-8 × Investment	1	9215.1	0.23	.633
Game × Investment	1	9162.0	4.53	.033
Condition × Investment	1	9208.4	34.82	< .001
RFQ-8 × Game × Condition	1	9071.0	5.89	.015
RFQ-8 × Game × Investment	1	9160.1	0.93	.335
RFQ-8 × Condition × Investment	1	9215.1	16.70	< .001
Game × Condition × Investment	1	9162.0	24.11	< .001
RFQ-8 × Game × Condition × Investment	1	9160.1	2.36	.125

× investment interaction was significant ($F(1, 155.9) = 4.50$, $p = .036$) while the 3-way with Game was not. The close resemblance between the DERS and PAI-BOR valence panels in Figure S3 reflects the strong empirical overlap between the two scales in this sample (Pearson r = 0.71), and the fact that at the mean of either trait the conditional slope reduces to the same main investment-by-Game effect.

We report this as a descriptive, exploratory pattern and not as evidence about mechanism, for three reasons. First, these analyses are exploratory and non-preregistered, with a family that spans multiple emotion outcomes, three trait measures, and several interaction structures, so the smaller p-values may not survive any standard multiple-comparisons correction. Second, the design does not include emotion ratings from the control group (who instead rated investment magnitude on the per-round grid), so pre-to-post change in the intervention group cannot be cleanly attributed to the intervention rather than to practice, fatigue, or carryover from the pre-game low investment. Third, both valence and arousal are self-reported on the same 2D grid; the design cannot adjudicate among candidate accounts of the directional asymmetry, such as a heightened evaluative response to investment fluctuations under emotion-dysregulation conditions, a response-style difference correlated with trait scores, or a trait-modulated adaptation effect across rounds.

5.2.2 Perceived selfishness and returns

Participants who rated their investor as more selfish returned less on average, and this perceived-selfishness → return link was robust across the sample and not moderated by trait. The headline effect held in every model ($F(1, 513.4) = 15.54$, $p < .001$ in the PAI-BOR model; consistent estimates with DERS and RFQ-8); none of the three trait measures moderated it (perceived-selfishness × PAI-BOR $F(1, 568.1) = 0.00$, $p = 1.000$; × DERS $F(1, 550.4) = 1.20$, $p = .274$; × RFQ-8 $F(1, 524.6) = 2.10$, $p = .148$); nor did the moderation vary by condition (the largest 3-way × Condition interaction reached $F = 3.03$, $p = .082$).

5.2.3 Post-low-investment retaliation

Adopting a post-low-investment retaliatory strategy (mean return below 30% at and after the low investment; 28.3% of participants) was not predicted by any questionnaire score (PAI-BOR OR = 0.94, $p = .733$; DERS OR = 1.04, $p = .824$; RFQ-8 OR = 0.95, $p = .791$), whereas the intervention roughly halved the odds (OR = 0.44, $p = .001$), reproducing the main effect.

That threshold is a behavioural proxy for “getting stuck”. We also ran the question directly on the latent states. We reused the locally-decoded trustee states from the main-text hidden Markov model (Figure 4.B).

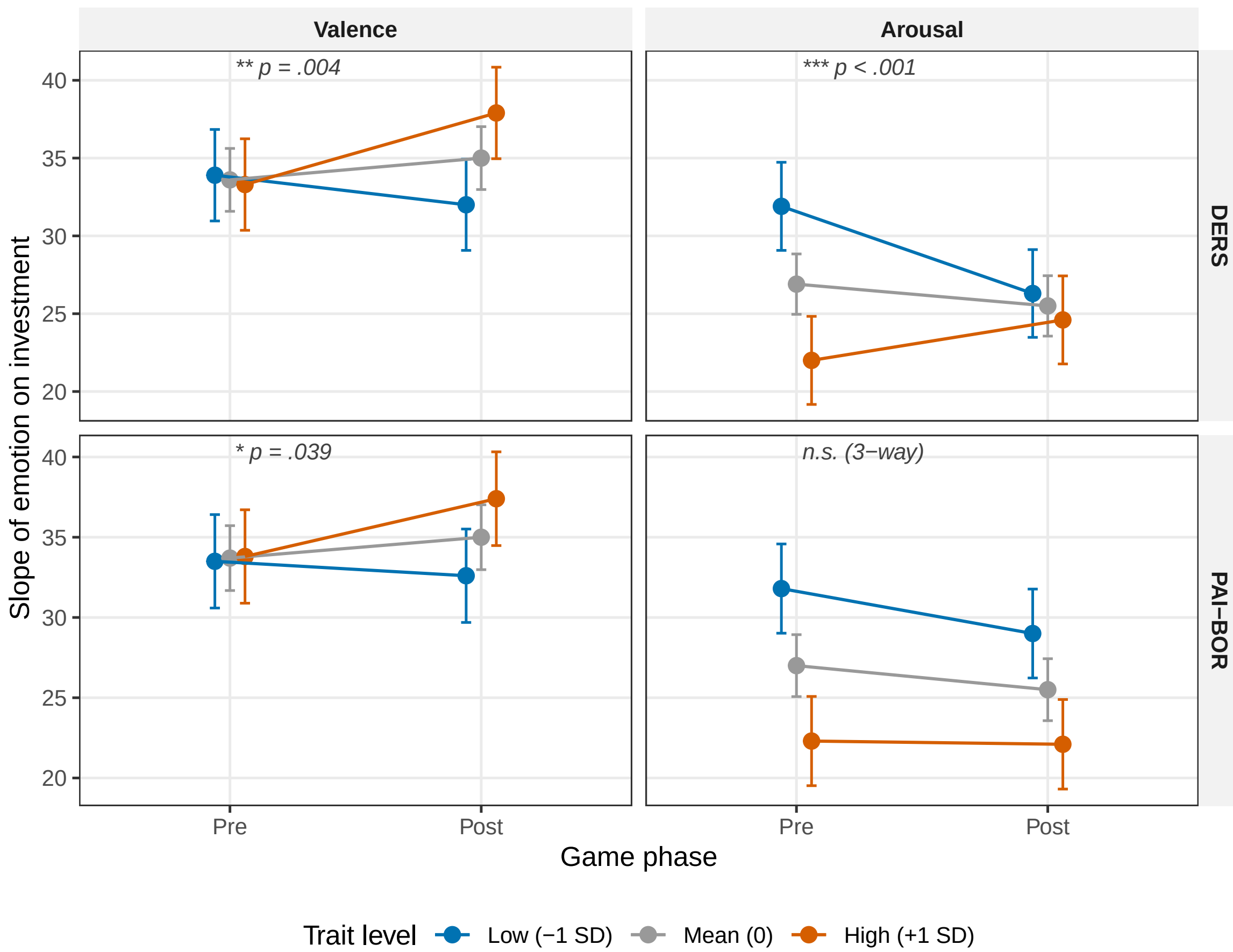

Figure S3: Simple slopes of immediately-reported emotion on investment received, in the intervention group, by game phase (Pre, Post) and trait level. Rows: trait moderator (DERS, top; PAI-BOR, bottom). Columns: emotion outcome (Valence, left; Arousal, right). Points are slope estimates from emmeans at low (-1 SD), mean (0), and high (+1 SD) of the standardised trait; error bars are plus or minus one standard error. Strip annotations report the omnibus emotion x trait x Game interaction (*** $p < .001$, ** $p < .01$, * $p < .05$, n.s. $p >= .05$); for arousal x PAI-BOR the 3-way is not significant but the 2-way trait x investment is ($F = 4.50$, $p = .036$).

For each participant we asked whether trait scores predict occupying the lowest-return state (State 1, the most retaliatory return policy) on the trial after the post-manipulation low investment (round 14). That round is the point at which the main text indexes getting stuck.

23.0% of participants occupied that lowest-return state at round 14. Adjusting each trait for Condition, none predicted membership of the state (PAI-BOR OR = 1.05, p = .729; DERS OR = 1.08, p = .563; RFQ-8 OR = 0.95, p = .700). The intervention, by contrast, reduced the odds of being stuck there (OR = 0.40, p = .001). The model-based test agrees with the behavioural proxy. Who remained in the most retaliatory latent state after the trust withdrawal was governed by the manipulation, not by pre-existing borderline, emotion-regulation, or mentalising traits. Like the other analyses in this section, this test is exploratory and not preregistered, and we report it as hypothesis-generating.

6 Hidden Markov model used to simulate the Investor's actions

The HMM assumes that the probability of each investment $I_t = 0, \ldots, 20$, at each trial t, conditional on the current state of the investor S_t, is dependent on an underlying normal distribution with mean μ_s and standard deviation σ_s. The probability of each discrete investment was determined from the cumulative normal distribution Φ, computing the probability of a Normal variate falling between the midway points of the response options. As responses were bounded at 0 and 20, we normalised these probabilities further by taking the endpoints into account. For instance, the probability of an investment $I_t = 2$ is defined as:

$$P(I_t = 2|S_t = s) = \frac{\Phi(2.5|\mu_s, \sigma_s) - \Phi(1.5|\mu_s, \sigma_s)}{\Phi(20.5|\mu_s, \sigma_s) - \Phi(-0.5|\mu_s, \sigma_s)}$$

Note that the denominator truncates the distribution between 0 and 20. To estimate the transition probability between states for the investor, a multinomial logistic regression model was fitted to the investor's data such as:

$$P(S_{t+1} = s'|S_t = s, X_t = x) = \frac{\exp(\beta_{0,s,s'} + \beta_{1,s,s'}x)}{\sum_{s''} \exp(\beta_{0,s,s''} + \beta_{1,s,s''}x)}$$

where $X_t = R_t - I_t$ is the net return to the investor with R_t the amount returned by the trustee and I_t is the Investment sent.

The advantage of this approach is that it does not require any a priori assumptions about the model features. The number of states, the policy conditional on the state, and the transition function between states can all be determined in a purely data-driven way. These HMMs can in turn be used to simulate a human-like agent playing the trust game. This agent may transition to a new state depending on the other player's actions and adopt a policy reflecting its state, thus simulating changes in emotional dispositions of human players during a repeated game. When the investor gains from the interaction, they become more likely to transition to a state where their policy is more "trusting" with generally higher investments. However, faced with losses, the investor is more likely to transition to a more cautious policy with generally lower investments. The policies and the transitions between states are sufficient to build an agent that reflects this type of adaptive behaviour and reacts to the trustee's action choices in a way that mimics a human player.

We estimated a three-state model for investor's behaviour, using maximum likelihood estimation via the Expectation-Maximisation algorithm as implemented in the `depmixS4` package for R (Visser & Speekenbrink, 2021). The model was estimated using investments from existing datasets of human dyads playing 10 rounds of the RTG with the same trustee. The dataset consisted of a total of 381 games from two data sources: First, a total of 93 repeated trust games with healthy investors and a mix of healthy trustees and trustees diagnosed with Borderline Personality Disorder (BPD) (King-Casas et al., 2008). The second source was from data collected as part of a project investigating social exchanges at the Montague lab at Virginia Tech and consists of 288 games.

7 Returns by round and agent investment received

The main text presents the behavioural effect starting with the low-investment response (Figure 2). For completeness, this section shows two descriptive views: the percentage return round by round, and the investment received from the simulated investor. Both are descriptive summaries (means with standard errors); the inferential tests are in the main text.

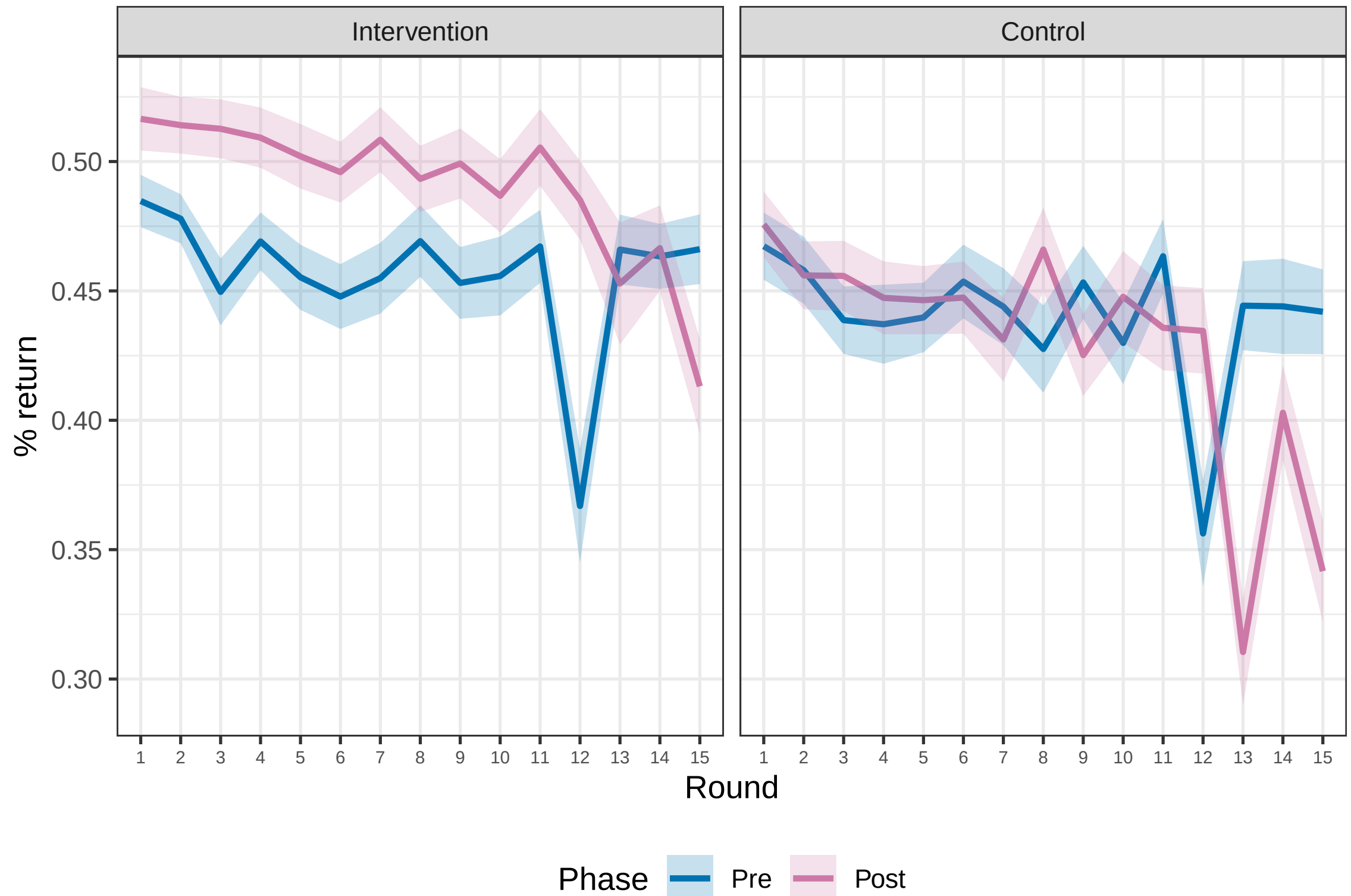

Figure S4: Percentage return by round and condition, shown separately for the pre-manipulation (blue) and post-manipulation (pink) games. Lines are means across participants and bands are plus or minus one standard error.

8 Mixed effects models of participants percentage returns

For trials before and after the programmed low investment, we model the percentage return (percentage of tripled investment returned to investor) using the same linear mixed-effects model used for all the rounds as described in the main text:

$$
\begin{aligned}
R_{ij} =& \beta_0 + \beta_1 \text{ (Condition)}_i + \beta_2 \text{ (Game)}_i + \beta_3 \text{ (Investment)}_i + \\
& \beta_4 \text{ (Condition} \times \text{Game)}_i + \beta_5 \text{ (Condition} \times \text{Investment)}_i + \beta_6 \text{ (Game} \times \text{Investment)}_i + \\
& \beta_7 \text{ (Condition} \times \text{Game} \times \text{Investment)}_i + b_{0j} + b_{1j} \text{ (Game)}_i + \epsilon_{ij}
\end{aligned}
$$

where:

- R_{ij}: percentage of tripled investment returned to investor for participant j in observation i
- β_0: intercept
- β_1: effect of Condition (intervention vs. control)
- β_2: effect of Game (RTG game pre vs. post-intervention)

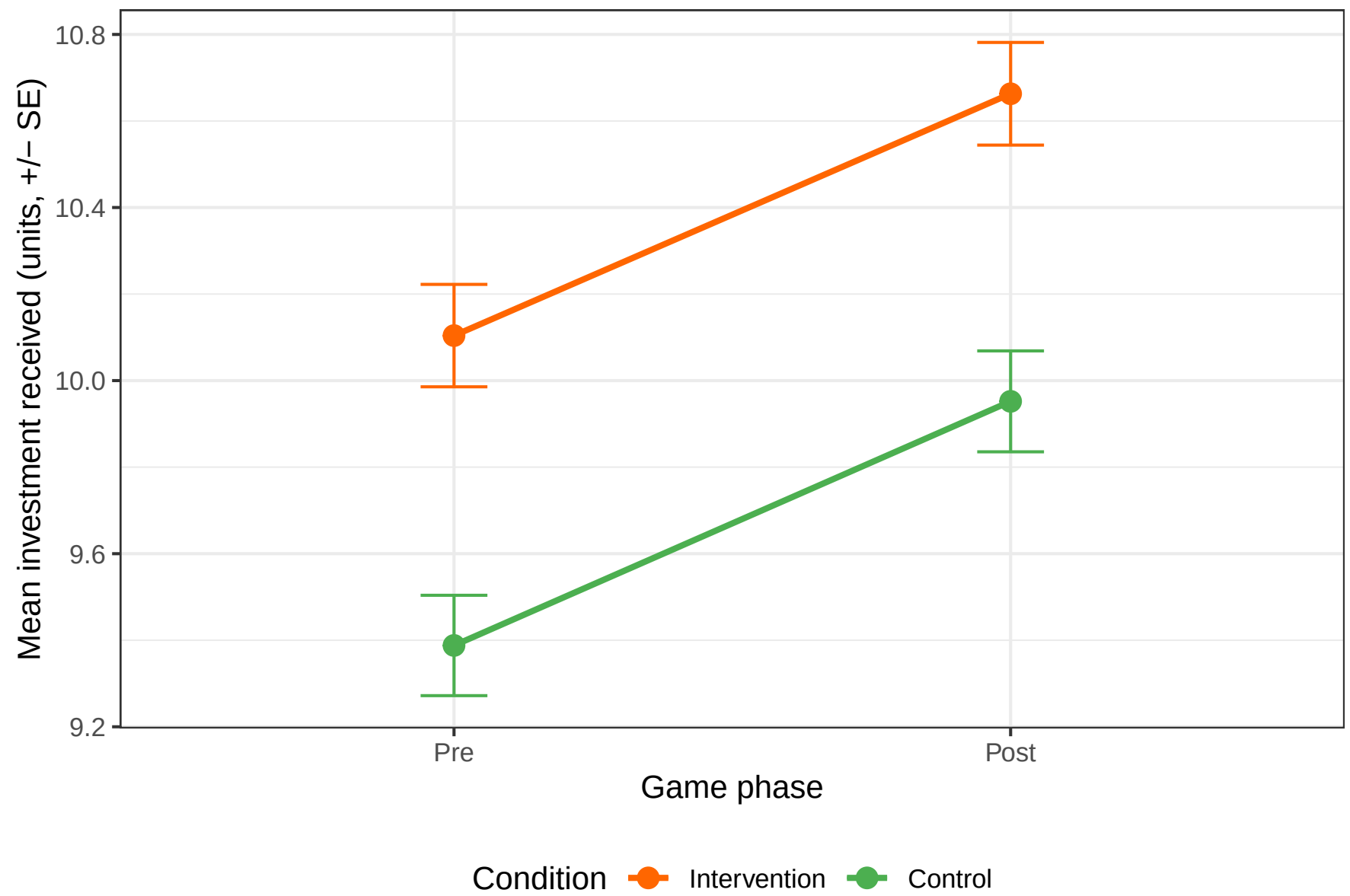

Figure S5: Investment received from the simulated investor by condition and game phase. Points are mean investment across all trustee rounds and error bars are plus or minus one standard error. The simulated investor sent more to the intervention group and more after the manipulation, following from the higher returns it received.

- β_3: effect of Investment
- β_4: interaction effect between Condition and Game
- β_5: interaction effect between Condition and Investment
- β_6: interaction effect between Game and Investment
- β_7: three-way interaction effect between Condition, Game and Investment
- b_{0j}: participant-wise random intercept for participant j
- b_{1j}: participant-wise random slope for Game for participant j
- ϵ_{ij}: error term for participant j in observation i

8.1 Mixed-effects model results for pre-low-investment returns

We fit the above linear mixed effects model to all trials prior to the pre-programmed low investment by the HMM investor (occurring on round 12 pre-manipulation, and round 13 post). A summary of results can be seen in Table S4.

Table S4: Summary of results of a mixed-effects model fitted to participants' returns restricted to pre-low-investment trials.

Term	Estimate	Std. Error	df	t value	Pr(>|t|)
(Intercept)	0.46	0.01	315.63	71.82	0.00
Game	-0.01	0.00	319.08	-3.26	0.00
Condition	0.02	0.01	315.63	3.08	0.00
Investment	0.02	0.00	7,041.28	11.58	0.00
Game:Condition	-0.01	0.00	319.08	-4.23	0.00
Game:Investment	0.00	0.00	6,877.90	-2.41	0.02
Condition:Investment	-0.01	0.00	7,041.28	-6.00	0.00
Game:Condition:Investment	0.01	0.00	6,877.90	4.15	0.00

8.2 Mixed-effects model results for post-low-investment returns:

In the same way, we fit the linear mixed effects model to all post-low-investment trials by the HMM investor, including the round on which the low investment occurs. A summary of results is presented in Table S5

Table S5: Summary of results of a mixed-effects model fitted to participants' returns restricted to post-low-investment trials.

Term	Estimate	Std. Error	df	t value	Pr(>|t|)
(Intercept)	0.43	0.01	338.49	51.37	0.00
Game	0.02	0.01	369.84	3.93	0.00
Condition	0.02	0.01	338.49	2.38	0.02
Investment	0.04	0.00	1,985.67	9.25	0.00
Game:Condition	-0.02	0.01	369.84	-3.54	0.00
Game:Investment	0.01	0.00	1,981.63	3.51	0.00
Condition:Investment	-0.01	0.00	1,985.67	-3.15	0.00
Game:Condition:Investment	0.01	0.00	1,981.63	1.79	0.07

9 Descriptive statistics and additional robustness checks

9.1 Descriptive statistics of analysis variables

The table below reports descriptive statistics for the analysis variables at the levels of the design: the individual (questionnaire scores, one value per participant) and the trial (investment received, percentage return, round). At the game-phase level the design is balanced, with 159 participants in each condition (intervention and control), each contributing a pre-manipulation and a post-manipulation game. Trial-level rows are over the 318 analysis participants; the percentage return is defined on the 9,372 trials where the investor sent a positive amount.

Table S6: Descriptive statistics of the analysis variables by level of the design. Individual-level rows are over the 318 participants (one value each); trial-level rows are over all trustee trials.

Variable	Level	N	Mean	SD	Min	Median	Max
PAI-BOR score	Individual	318	22.47	10.62	0	22.00	60
DERS score	Individual	318	80.36	22.49	35	77.00	144
RFQ-8 (certainty)	Individual	318	1.22	0.86	0	1.17	3
Investment received (units)	Trial	9540	10.02	5.76	0	10.00	20
Percentage return	Trial	9372	0.45	0.19	0	0.49	1
Round number	Trial	9540	8.00	4.32	1	8.00	15

9.2 Distribution of percentage return and a binomial robustness re-fit

We checked whether the bounded, skewed distribution of percentage return is a problem for the Gaussian mixed model used in the main text. The percentage return lies in the unit interval and is left-skewed (n = 9372; mean 0.454, SD 0.191; skew -0.54), with 6.3% of trials at exactly 0, 1.3% at exactly 1, and 92.4% in between. Residuals from the main-text model were approximately symmetric (skew 0.06) but heavy-tailed (excess kurtosis 6.06), so the concern is legitimate. As a principled robustness check we refit the model as a binomial generalised linear mixed model, treating the amount returned as a count out of the tripled investment, with the same fixed-effect structure. In the headline model (participant random intercept, which converged cleanly), the key effects were unchanged and, if anything, sharper: the Condition × Game interaction (z = 10.86, p < .001), the three-way Game × Condition × Investment interaction (z = -6.98, p

$< .001$), and the Investment effect (z = 10.14, $p < .001$) all held, while the Condition × Investment term remained non-significant (z = 0.41, p = .68). A secondary binomial model with a by-game random slope agreed (Condition × Game z = 4.59, three-way z = -6.50, both $p < .001$). The Condition main effect was not significant on the logit scale (z = 1.36, p = .17), as expected given the change of link and coding; the substantive claims live in the interactions, which replicate. The conclusions therefore do not depend on the Gaussian approximation.

9.3 Absolute versus relative-deviation coding of the investment

We also asked whether returns are governed by the absolute size of the current investment or by its deviation from recent expectation (Palminteri et al., 2015). Using the trials from round two onward (8739 observations, 318 participants), we compared three single-predictor models of percentage return with the same fixed and random structure (maximum likelihood): the absolute investment, the change from the previous round, and the deviation from the expanding mean of prior rounds. The absolute-investment model fit best (AIC -7897.8, versus -7875.7 for deviation-from-expectation and -7731.0 for change-from-previous). Adding deviation-from-expectation to a model that already contained absolute investment did not improve fit (likelihood-ratio $\chi^2(1)$ = 0.26, p = .61). With both standardised predictors in one model, the absolute investment dominated (β = 0.026, t = 5.66, $p < .001$) while the deviation term was negligible (β = 0.002, t = 0.47, p = .64). Returns are therefore governed by the absolute size of the current investment, not by its drop relative to recent history. The pre-programmed low investment still functions as a violation because a fixed value of two units is absolutely very low, not because it is low relative to expectation. Expectation here is operationalised as the expanding mean of prior rounds, which is one reasonable choice.

9.4 Retaliator-threshold robustness

The main text classifies a participant as a retaliator if their mean percentage return at and after the pre-programmed low investment falls below 30%. The 30% cut-off is a convenience, so we repeat the pre-to-post McNemar test at 25%, 30%, and 35%. The control group's retaliator rate rises significantly at every threshold, whereas the intervention group's does not change at any, so the result does not depend on the particular cut-off.

Table S7: Retaliator-threshold robustness. A retaliator is a participant whose mean percentage return at and after the pre-programmed low investment falls below the stated threshold. Pre and Post give the percentage of retaliators in the pre- and post-manipulation games; McNemar is the chi-square statistic (degrees of freedom in parentheses) for the within-group pre-to-post change. The control-group increase is significant at every threshold, whereas the intervention group does not change at any.

Threshold	Group	Pre (%)	Post (%)	McNemar (df)	p
25%	Control	14.5	29.6	13.22 (1)	$< .001$
30%	Control	20.8	36.5	14.05 (1)	$< .001$
35%	Control	26.4	47.2	21.79 (1)	$< .001$
25%	Intervention	10.7	13.8	0.55 (1)	.458
30%	Intervention	17.0	20.1	0.43 (1)	.511
35%	Intervention	26.4	31.4	1.11 (1)	.291

9.5 The control group's per-round rating is veridical

The control group's per-round rating is the speed-and-magnitude evaluation defined in the Methods; it is not an emotion measure. We tested whether it reflects the partner's actual behaviour or an independent subjective drift by relating each control participant's perceived-magnitude rating (rescaled to the unit interval) to the objective investment received. The rating tracked the objective investment closely (r = .88 across control trials), including the sharp fall at the pre-programmed low investment (mean rating 0.11 at that

round, against 0.51 on the other rounds). In a mixed-effects model with a participant random intercept, the objective investment was an overwhelming predictor of the rating (standardised $b = 0.27$, $t = 147$), and the round trend, already small, shrank from -0.04 to -0.01 once the objective investment was included. The rating is therefore veridical: it registers how much the investor actually sent, and its change over the game reflects the investment trajectory itself, not a rating-induced drift.

This bears on the possibility that repeatedly rating investment magnitude made control participants more self-interested. We find no independent rating drift, and the control group's returns did not decline monotonically; like the rating, they were stable before the manipulation and fell specifically at the pre-programmed low investment (the retaliation reported in the main text). Both the rating and the behaviour track the objective low investment rather than a self-generated trend toward self-interest. Because the two conditions rated different dimensions and there is no no-grid control, an effect of the grid content cannot be fully excluded, and we retain this as a limitation in the main text; the present evidence does not support the reverse-direction account.

9.6 Declared change after the intervention and its relation to behaviour

During the intervention session, participants first stated how they would respond to a hypothetical low investment, then read the educational slide and reported in free text whether it would change that response. We coded the 159 usable responses by whether each declared a shift toward the cooperative, trust-coaxing strategy, relative to the participant's stated pre-slide intention, or kept that plan. Coding followed a fixed codebook applied by a large language model (Claude Opus 4.7, Anthropic) in two independent passes, the second blind to the first; the passes agreed on 152 of the 159 responses (Cohen's $\kappa = .90$, 95.6% agreement). A co-author adjudicated the remaining seven, resolving all but one and reporting that genuinely ambiguous case under both codings. By the adjudicated coding, 35.2% of participants (56 of 159) declared a shift toward cooperation.

Declared change tracked behaviour. We defined actual change as each participant's difference, between the pre- and post-manipulation games, in mean percentage return over the low-investment window (the pre-programmed low investment and the two rounds after it). Participants who declared a shift showed a more positive change than those who did not (M = 0.067 vs. -0.016; Welch $t(112.9) = -2.75$, $p = .007$; Wilcoxon $p = .007$; point-biserial $r = .21$). The association was not an artefact of coding: it held under the blind replicate, under exclusion of the seven adjudicated cases, and under the least favourable of every possible relabeling of those seven cases (largest $p = .015$). Declared intention thus predicted a modest but reliable behavioural shift, and the majority who declared no change accords with the individual differences identified by the hidden Markov model analysis.

10 Subgroup Analysis by Belief About Opponent

At debrief, participants were asked whether they thought the investors they faced were human or computer-controlled. We coded responses into three categories based on keyword matching: (1) believed machine (responses mentioning "machine," "computer," "bot," "program," or "AI" without human references), (2) believed human (responses mentioning "human," "people," or "person" without machine references), and (3) unsure (responses expressing uncertainty or mentioning both human and machine). The distribution was: 56% believed machine, 22% believed human, and 22% were unsure (44% human or unsure combined).

To examine whether the intervention effect depended on participants' beliefs about the nature of their opponent, we included belief category as an additional factor in our mixed-effects model. Belief category (`belief_f` in the analysis) was coded from each participant's free-text response about whether they had faced a human or a computer, into three levels: believed-machine, believed-human, and unsure (the keyword-based coding rule is the same as in the main text). Table S8 shows the distribution of belief categories by experimental condition; randomisation successfully balanced beliefs across conditions.

Table S8: Distribution of belief about opponent by experimental condition

Condition	Believed Machine	Believed Human	Unsure
intervention	90	35	34
control	88	36	36

10.1 Full ANOVA Results with Belief Moderation

Table S9 presents the full ANOVA results for the mixed-effects model including belief category and all interactions.

Table S9: ANOVA results for the mixed-effects model of percentage return with belief moderation

Effect	df1	df2	F	p
Game	1	314.6	1.86	.174
Condition	1	312.7	3.98	.047
Investment	1	325.2	33.52	< .001
Belief	2	312.7	3.17	.044
Round	1	8651.6	106.71	< .001
Low-investment round	1	8646.0	23.03	< .001
Game × Condition	1	314.4	21.49	< .001
Game × Investment	1	8916.6	0.29	.590
Condition × Investment	1	307.3	5.28	.022
Game × Belief	2	314.4	0.08	.926
Condition × Belief	2	312.7	1.65	.194
Investment × Belief	2	307.9	0.69	.503
Game × Condition × Investment	1	8917.0	19.68	< .001
Game × Condition × Belief	2	314.4	0.80	.451
Game × Investment × Belief	2	8913.3	0.50	.607
Condition × Investment × Belief	2	307.9	0.33	.722
Game × Condition × Investment × Belief	2	8913.7	0.26	.769

The critical tests are the interactions involving belief category. Neither the three-way Condition × Game × Belief interaction ($F(2, 313.3) = 0.50$, $p = .61$) nor the four-way Condition × Game × Investment × Belief interaction ($F(2, 8873.5) = 0.26$, $p = .77$) was significant. This indicates that the intervention effect on cooperative behaviour did not depend on whether participants believed they were interacting with a human or machine opponent.

10.2 Condition Effects by Belief Category

Table S10 shows the intervention versus control contrast within each belief category during the post-intervention phase.

10.3 Pre-Post Change by Condition and Belief Category

Table S11 shows the pre-to-post change in cooperation within each combination of condition and belief category.

The pattern of results was consistent across belief categories: intervention participants increased cooperation from pre to post, while control participants either decreased or showed no change. Among participants who believed they were facing a human opponent, the intervention group increased cooperation ($p = .008$) while

Table S10: Intervention vs. Control contrast in percentage return in the post-intervention game, by belief category

Belief Category	Estimate	SE	df	t	p
Believed Machine	0.079	0.019	313.7501	4.20	$<$ .001
Believed Human	0.056	0.030	313.9179	1.88	.060
Unsure	0.022	0.030	311.8342	0.73	.467

Table S11: Pre-to-post change in percentage return by condition and belief category

Condition	Belief	Estimate	SE	df	t	p
intervention	Believed Machine	0.031	0.009	309.3793	3.35	$<$ .001
control	Believed Machine	-0.011	0.010	323.2315	-1.20	.233
intervention	Believed Human	0.040	0.015	316.1006	2.66	.008
control	Believed Human	-0.030	0.015	311.6046	-2.05	.041
intervention	Unsure	0.026	0.015	309.7296	1.70	.090
control	Unsure	-0.010	0.015	316.8995	-0.69	.488

the control group decreased cooperation ($p = .04$). The pattern is consistent with the same effect operating in the believed-human subgroup, with reduced power in the smaller subgroups; the non-significant three-way interaction reported above is the formal moderation test.

11 Hidden Markov models to analyse participants returns

To model participants' returns in the RTG across games and conditions, we fit various hidden Markov models to participants returns. The response function is modelled as a discretised Gaussian distribution that takes into account what proportion the trustee would ideally like to return, and what returns are possible given the investment. For instance, if the investor sends an amount of 2, the trustee would receive 6 and they can send back any amount between 0 and 6. We assume that the response is a distribution over the possible proportions that can be calculated from these possible returns, i.e. $\{0, 1/6, 2/6, ..., 1\}$. The model assumes an underlying Normal distribution for each possible proportional return, predicting the probability of each via the cumulative Normal distribution with cut-off points set halfway between the proportions (e.g. the probability of returning 1/6 is determined as the probability of returning anything between 1/12 and 3/12). The transition between states is assumed to depend on the investment through a multinomial logistic function such as:

$$P(S_{t+1} = s' | S_t = s, X_t = x) = \frac{\exp(\beta_{0,s,s'} + \beta_{1,s,s'}\ x + \beta_{2,s,s'}\ d)}{\sum_{s''} \exp(\beta_{0,s,s''} + \beta_{1,s,s''}\ x + \beta_{2,s,s''}\ d)}$$

where x is a variable representing the investment received, d is a dummy variable to characterise the group that the participant belongs to. We define four contrast codes for these dummy variables: pre-post (comparing pre and post games), post-coax (compares the post-intervention group to all others), post-control (compares the post-control group to all others) and full-contrast (a three level dummy variable: post-intervention compared to post-control and all pre games).

Table S12: Comparison of the HMM-full models fit assuming the latent number of states is between 2 and 7. Using BIC, the 6 state model fits best.

Number of states	logLikelihood	df	AIC	BIC
2	-24,921	18	49,879	50,008
3	-23,032	46	46,157	46,486
4	-22,244	86	44,660	45,276
5	-21,965	138	44,207	45,195
6	-21,635	202	43,673	45,120
7	-21,486	278	43,528	45,519

As seen in Table S12, using the BIC, the 6 state HMM-full model fits best. Since we only fit models between 2 and 7 states, it is possible that models with a higher number of states could fit the data better. We decided to stop at 7 states for computational cost reasons and because the interpretation of models with a higher number of states becomes complex.

11.1 Transition function of HMM model describing participant's returns

Using the HMM-full 6 state model, we can plot the transition probabilities of the model for pre and post-intervention as well as pre- and post-control manipulation separately (Figure S6). This allows us to see how the transition probabilities between latent states change across Games and Condition.

11.2 Validation: posterior predictive check and recovery

To verify that the participant-return HMM reproduces the observed behaviour and that its parameters can be recovered, we ran two checks. The first is a posterior predictive check: we treated the fitted six-state HMM-full model as the data-generating process, simulated 200 full datasets from it, and compared the simulated mean percentage return against the observed mean, both round by round within each Condition and Game cell and for the four Condition by Game means. The second is a recovery analysis: over 100 replicates, we simulated full datasets from the fitted model and refitted the six-state model to each. We then assessed two kinds of quantity: the behavioural quantities the manuscript interprets (the ordered state return-policies and the decoded state occupancy), aligned to the generating states by their return policies before comparing; and the raw emission and transition coefficients, compared element by element.

The posterior predictive check shows the model reproduces the behavioural structure well. Across the 60 round-by-Condition-by-Game cells, simulated means tracked observed means with a mean absolute error of 0.014 (root mean squared error 0.018), and 51 of 60 observed cell means (85 percent) fell inside the 95 percent predictive interval. The four Condition by Game means were reproduced with a mean absolute error of 0.005; three of the four lay inside the predictive interval, the exception being the intervention post-manipulation cell, which the model under-predicts by about 0.010 (it slightly smooths the sharpest post-withdrawal rise). Figure S7 shows the observed round-by-round means against the simulated predictive band.

Behavioural recovery (the quantities the manuscript interprets). These recover well. Each latent state's expected return recovers to a mean absolute error of 0.012, and the ordering of states from lowest to highest return is preserved. Decoded state occupancy matches the generating state on 79 percent of trials (mean occupancy error 0.035), and the post-withdrawal occupancy values reported in the main text recover to within about one percentage point.

Parameter recovery (the raw coefficients). These do not recover element by element: the fitted emission and transition coefficients correlate only weakly with their generating values, and some transition probabilities

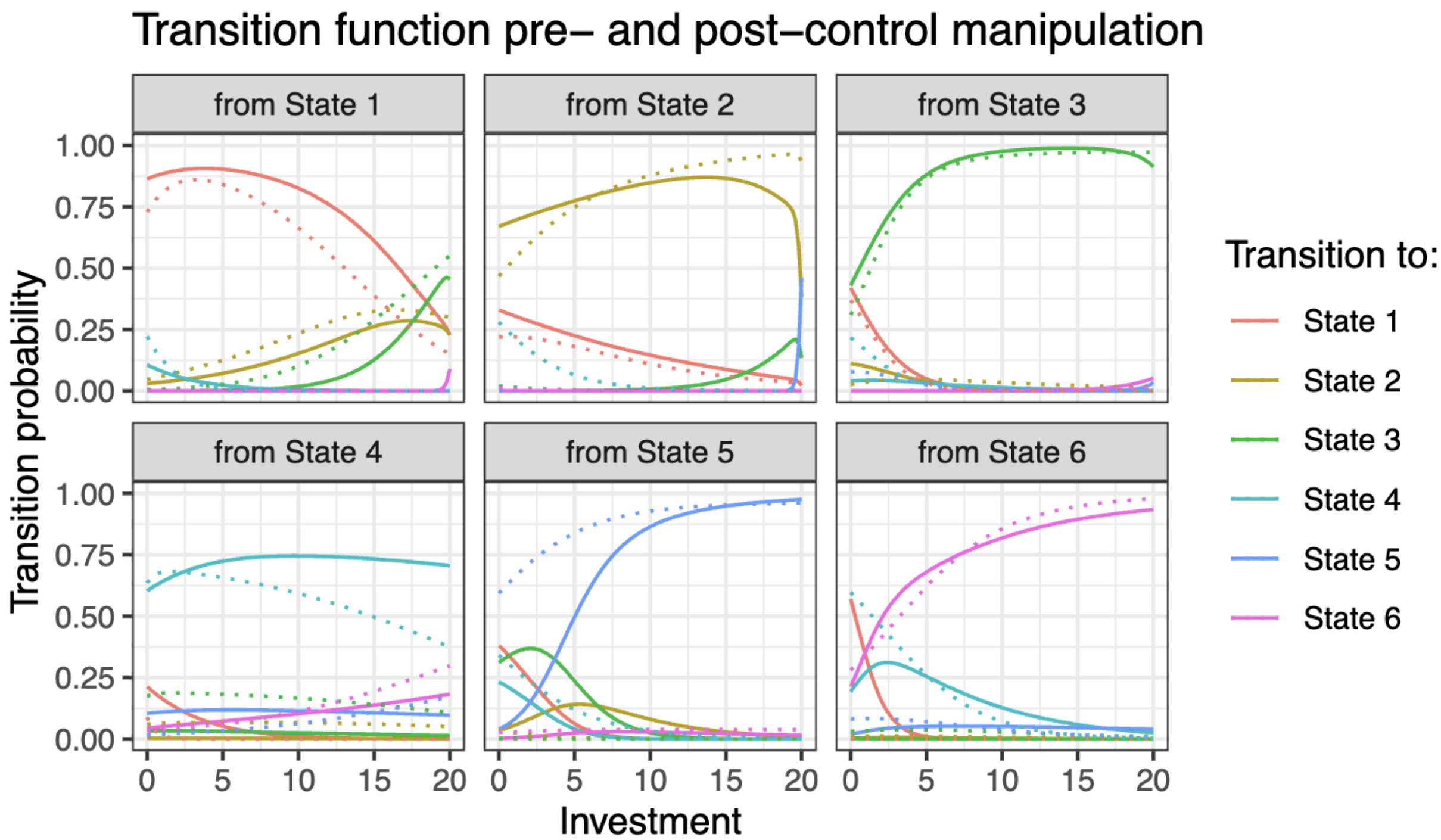

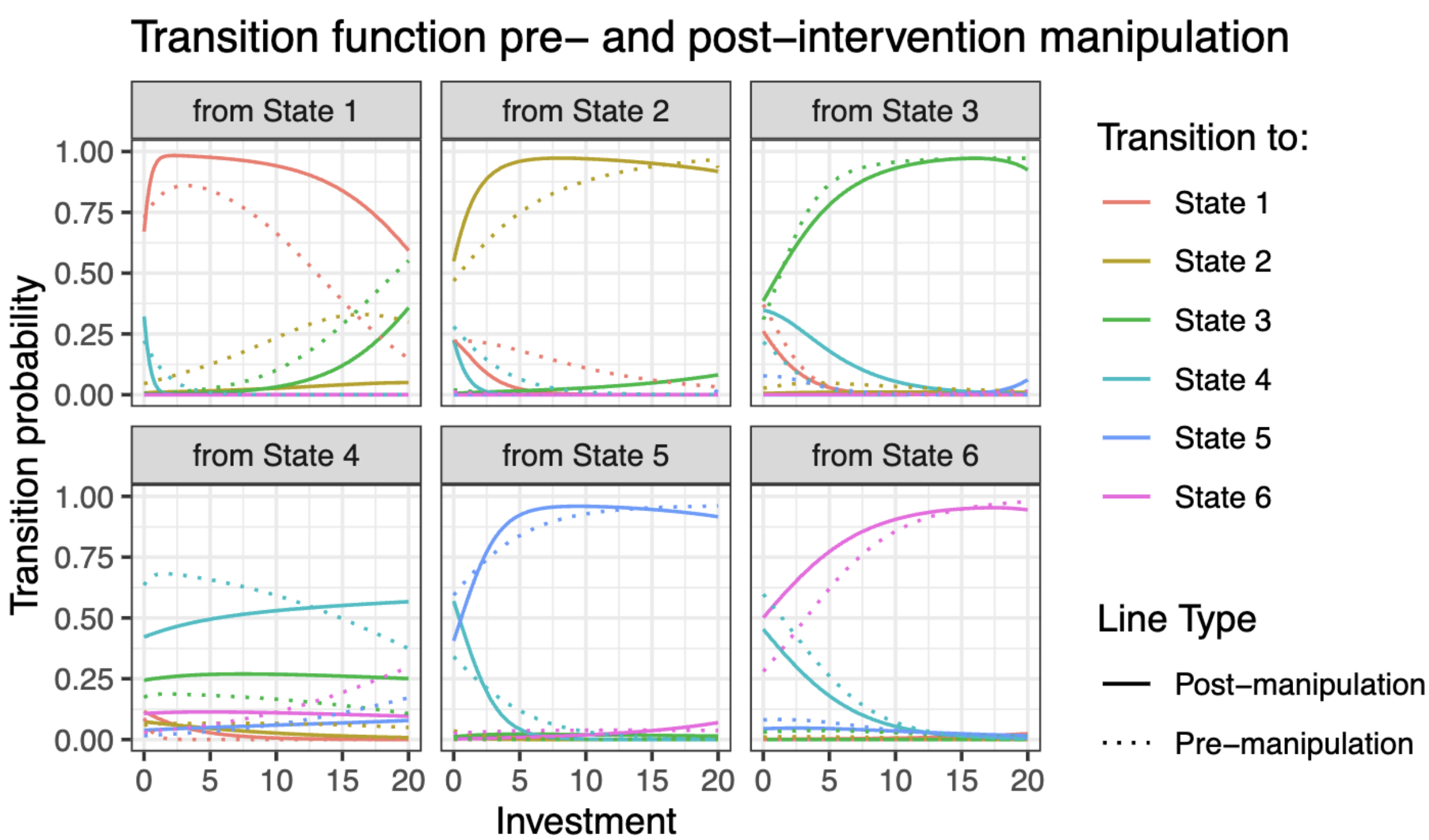

Figure S6: Transition function for the HMM-full trustee model. Each panel represents the state transitioned from. For instance the upper left panel represents all transition probabilities from state 1. The colours of the lines within each panel indicate the state transitioned to. For instance, in the upper left panel, the red line represents the transition probability to state 1 as a function of the received investment. In this case this is also the probability of staying in state 1 since we are in the state 1 panel. Solid lines show estimated transition probabilities post-manipulation while dotted lines show the same probabilities prior to the manipulation

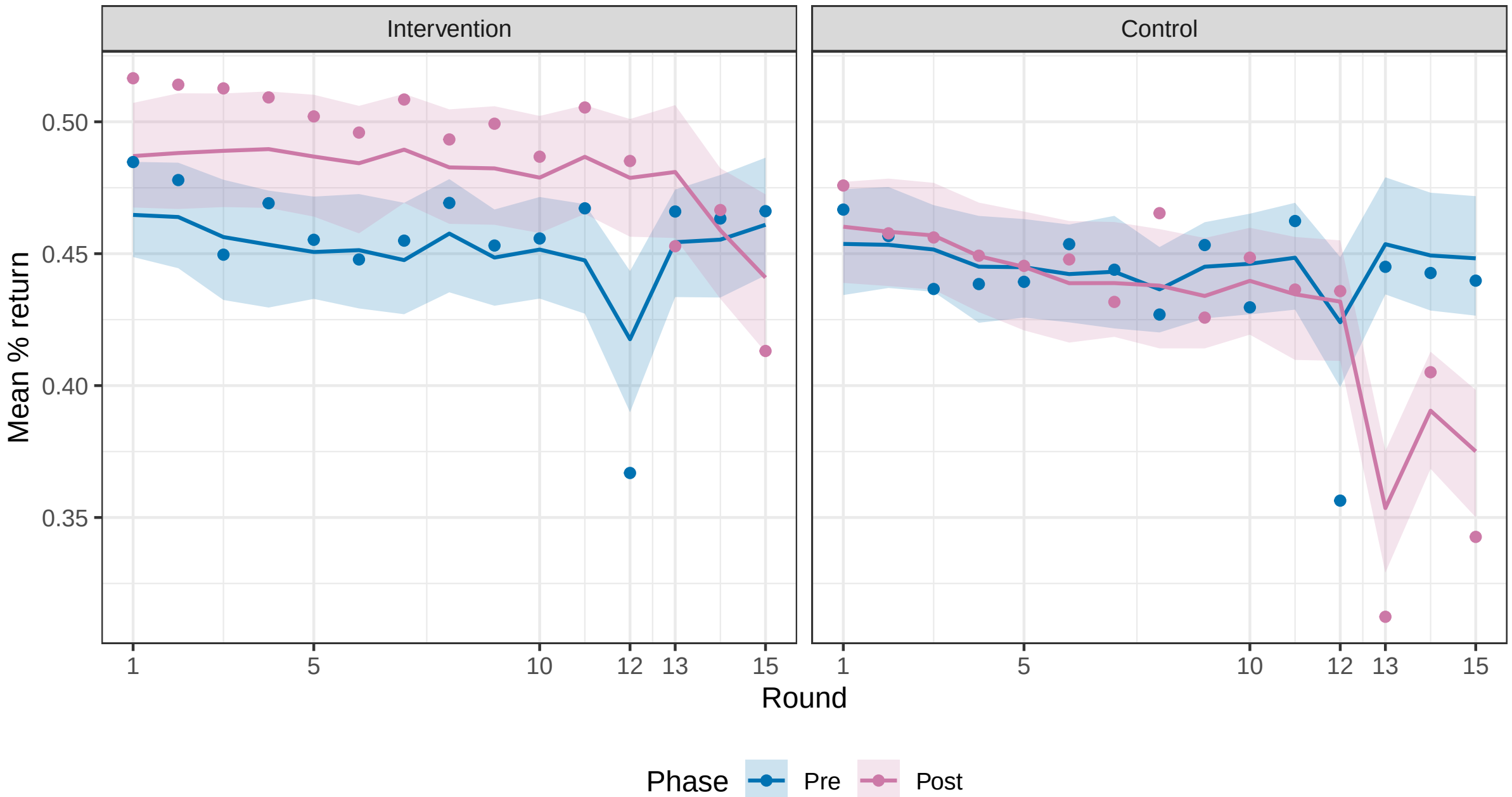

Figure S7: Posterior predictive check for the participant-return HMM. Points are observed mean percentage return by round; the shaded band is the 95 percent interval of the simulated mean across 200 datasets generated from the fitted six-state HMM-full model; the line is the simulated mean. Pre-manipulation game in blue, post-manipulation game in pink, by condition. Observed means fall inside the predictive band in 51 of 60 round-by-condition-by-game cells.

are off by 0.10 to 0.16 (mean transition-probability error 0.059). This is expected rather than a model failure. Hidden Markov model parameters are identified only up to a relabelling of the states: permuting the state labels leaves the likelihood unchanged (label switching), so a coefficient-by-coefficient comparison conflates genuine estimation error with arbitrary label permutations (Frühwirth-Schnatter, 2006). With six states and investment-dependent transitions the likelihood is also multimodal and weakly curved in the latent parameters, so many different parameter sets imply almost the same observable behaviour.

Taken together, the model reproduces the observed behaviour, and the quantities the manuscript interprets, the ordered return policies and the decoded post-withdrawal occupancy, recover well; the individual emission and transition parameters are not separately identifiable. We therefore interpret the HMM descriptively, in terms of state return-policies and occupancy, rather than treating individual parameters as precise mechanistic estimates.

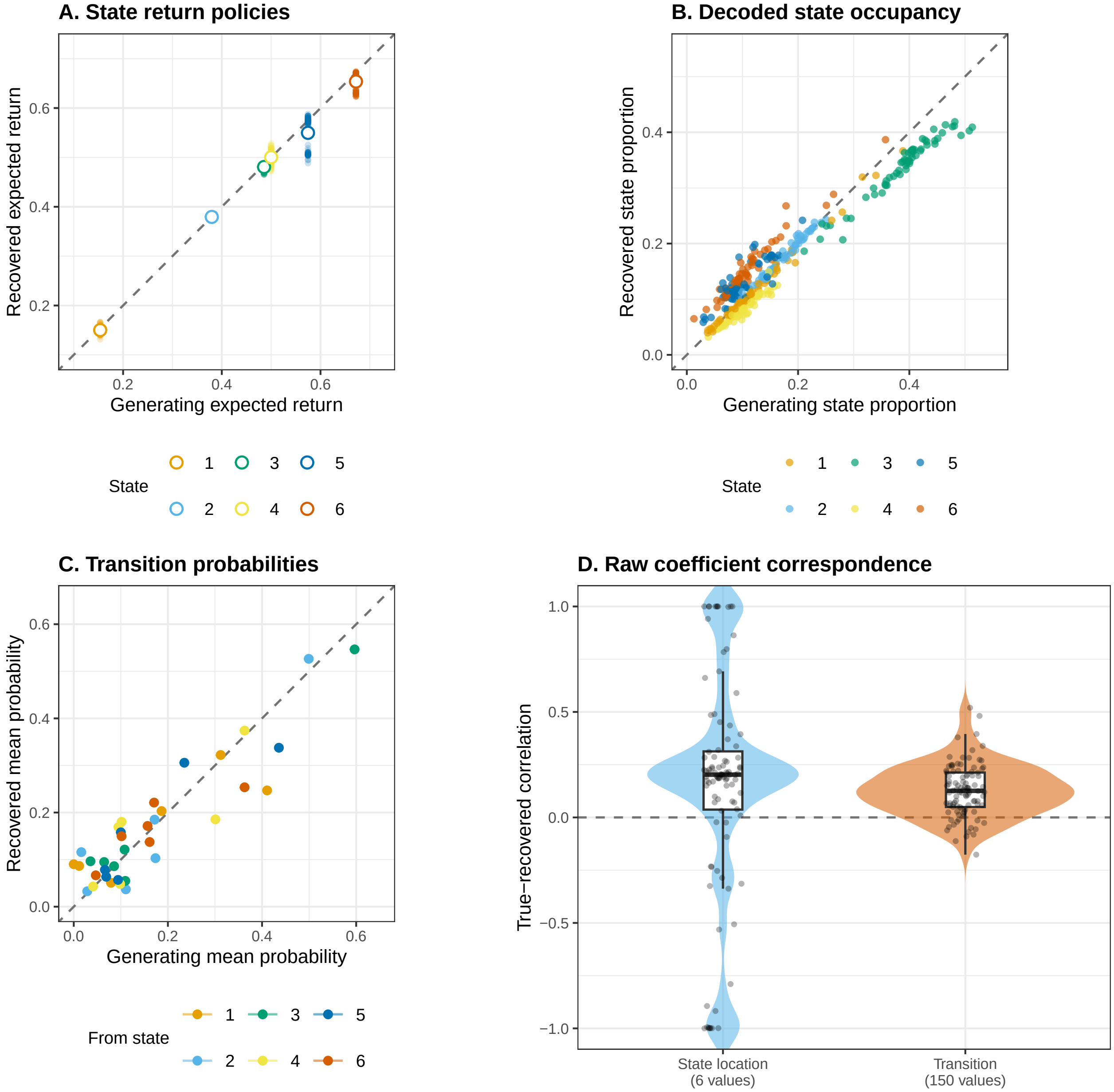

Figure S8: Recovery of the participant-return hidden Markov model across 100 simulated datasets. In Panels A-C, the diagonal represents perfect recovery. Panel A shows the expected return for each of the six latent states; small points are individual refits and larger outlined points are state means. Panel B shows decoded state occupancy, averaged across replicates within each Condition by Game by round by state cell. Panel C shows mean transition probabilities between states; error bars are 95 percent intervals across replicates. Panel D shows the per-replicate correlations between the generating and recovered raw state-location and selected transition coefficients.

References

Axelrod, R., & Hamilton, W. D. (1981). The Evolution of Cooperation. *Science*, *211*(4489), 1390–1396. https://doi.org/10.1126/science.7466396

Berg, J., Dickhaut, J., & McCabe, K. (1995). Trust, Reciprocity, and Social History. *Games and Economic Behavior*, *10*(1), 122–142. https://doi.org/10.1006/game.1995.1027

Frühwirth-Schnatter, S. (2006). *Finite Mixture and Markov Switching Models*. Springer. https://doi.org/10.1007/978-0-387-35768-3

King-Casas, B., Sharp, C., Lomax-Bream, L., Lohrenz, T., Fonagy, P., & Montague, P. R. (2008). The Rupture and Repair of Cooperation in Borderline Personality Disorder. *Science*, *321*(5890), 806–810. https://doi.org/10.1126/science.1156902

Palminteri, S., Khamassi, M., Joffily, M., & Coricelli, G. (2015). Contextual modulation of value signals in reward and punishment learning. *Nature Communications*, *6*(1), 8096. https://doi.org/10.1038/ncomms9096

Preti, E., Richetin, J., Poggi, A., & Fertuck, E. (2023). A Model of Trust Processes in Borderline Personality Disorder: A Systematic Review. *Current Psychiatry Reports*, *25*(11), 555–567. https://doi.org/10.1007/s11920-023-01468-y

Visser, I., & Speekenbrink, M. (2021). *depmixS4: Dependent Mixture Models - Hidden Markov Models of GLMs and Other Distributions in S4*.
