# Supplementary material for: (Re)building Cooperation: Effects of a Cognitive Intervention on Cooperative Behaviour in Games
Source: Comput Psychiatr. 2026 Sep 16;10(1):184–201. doi: 10.5334/cpsy.178 (PMC13588210; doi:10.5334/cpsy.178)
Supplement: Peer review history

# Reviewer report: Round 1, Reviewer A

**DOI:** 10.5334/cpsy.178.r1

**Authors:** Reviewer: A

## submission-comments

The manuscript entitled “(Re)building cooperation: Effects of a cognitive intervention on cooperative behaviour in games” uses economic games, behavioral data, and computational modeling to examine changes in cooperative behavior in response to an intervention derived from behavior therapy.

In general, the manuscript seems to fit well with the Computational Psychiatry Journal. Systematically testing behavioral interventions combined with computational modeling is a promising avenue for the field of computational psychiatry.

Despite the overall positive impression, I have several major concerns.

(1) Results description

I found it difficult to follow the results during my first pass through the manuscript. One issue was that the figure subpanels are in a different order than the results description (e.g., first 2.A, then 2.C., but 2.B is mentioned much later). Also, in some paragraphs, there are no links to the figures (e.g, lines 245-247, 253-258). To address this, paragraphs on one type of analysis should be merged. For example, on page 8, paragraphs 2 and 3 both concern the same type of analysis. Paragraph 2 describes the main effect of condition, paragraph 3 describes the respective post-hoc contrasts. Currently, only paragraph 3 refers to Fig. 2.C. In other cases, analyses are not plotted or statistically quantified. Another example is paragraph 8 and 9 (page 8), where the authors could add figure panels, and the last paragraph, where the authors should add relevant statistics.

Finally, in some cases, the paragraphs (such as lines 271-272) are quite short, and the motivation behind the analyses is initially somewhat unclear. I suggest that the authors walk the readers more explicitly through the results, with a short motivation behind the analyses and their implications for the hypotheses.

(2) HMM description

The general background of HMMs is very briefly introduced. For example, transition and emission parameters are insufficiently explained and defined. Another example is that the concept of a latent state, in particular with respect to the current task, is only superficially introduced. The authors should certainly refer to Figure 1.C and 1.D in the HMM results section as well (page 11), not just in the methods. Therefore, if the goal is that readers with a background in clinical psychology and not primarily computational modeling can smoothly follow the manuscript, then this section needs careful revision. On the other hand, also readers who want to understand the details of the model don’t find the relevant information in the main manuscript. The Methods currently don’t formally introduce the model; this is completely outsourced to the supplement.

(3) HMM validation

Moreover, the manuscript currently lacks sufficient HMM validation analyses. I could not find a proper posterior predictive check indicating how well the model captures participant behavior in general. Does the model capture the behavior shown in Fig. 2.A? Would it be able to reproduce Fig. 2.C?

Moreover, I was wondering whether parameters can be accurately recovered, and suggest that the authors add a systematic parameter recovery analysis.

(4) Discussion of potential demand or instruction effects

On page 13 (lines 410-421), the authors argue that demand or instruction effects are unlikely due to (1) the way the intervention was framed, (2) the presence of individual differences, (3) the control condition, and (4) the absence of an effect in the prisoner’s dilemma.

In my opinion, all of these points can’t rule out an instruction effect. It is not unlikely that participants simply followed the instruction that clearly explained why cooperation makes sense in the task (Fig. 1.B). The presence of individual differences does not rule this out; virtually any task with clear instructions yields individual differences. Moreover, the control condition did not provide this instruction, and it is plausible that this straightforwardly explains why cooperative behavior was different between the conditions. Finally, the fact that the authors did not find a similar effect in the prisoner’s dilemma can be interpreted in terms of a quite specific instruction effect in the RTG rather than a general insight that participants should behave cooperatively across all kinds of situations in their daily life. Therefore, I think that the authors should revise their discussion of the possibility of instruction effects and what this would imply for generalizable effects of DBT interventions.

## Recommendation

Revisions Required

# Reviewer report: Round 1, Reviewer C

**DOI:** 10.5334/cpsy.178.r2

**Authors:** Reviewer: C

## Recommendation:

Accept with Minor Revision

## Comments/Explanation (required). Please include references to any minor or major revisions required, as well as addressing any issues with language clarity. Authors will see these comments:

1. General comments and summary of recommendation. The manuscript fits within the journal’s scope, addressing social decision-making and computational/experimental approaches to psychopathology.
2. Content. The study is original and methodologically rigorous, with a strong experimental design (RCT, HMM agents, mixed models).
3. Structure and argument. The manuscript structure can be improved in terms of conceptual framework (introduction) and integration of the main findings into the very same coherent picture (discussion). The abstract is accurate but could emphasize the key mechanism more explicitly.
4. Figures/tables. Figures are relevant and informative.
5. Formatting. The manuscript generally adheres to standard formatting guidelines.
6. Language. The manuscript is clearly written, and I appreciated the effort the authors invested in making HMM accessible. Some sections are conceptually dense rather than linguistically unclear.

This manuscript reports an online randomized controlled study testing whether a brief DBT-inspired cognitive intervention can reduce retaliatory responding and promote more resilient cooperation after a programmed trust withdrawal in a repeated trust game. Participants acted as trustees across two repeated trust game phases, with the intervention delivered between phases, and then completed a repeated prisoner’s dilemma to test transfer. The investor in the trust game was a computerized HMM-based agent trained on human data, and the authors analysed participant behaviour using both mixed-effects models and hidden Markov models. The main claim is that the intervention increased returns in the post-intervention trust game, especially around the programmed low-investment trial, without altering self-reported emotional responses, and that this effect did not transfer to the repeated prisoner’s dilemma. The paper is interesting, methodologically ambitious, and potentially valuable as a proof-of-concept study on trust repair and experimentally tractable psychotherapy mechanisms.

I found the paper interesting and potentially publishable, and I think the core result is promising. In particular, the attempt to isolate a specific behavioural effect of a brief intervention at the moment of trust withdrawal is novel and worth pursuing. The HMM-agent approach is also creative and provides strong experimental control.

At present, there are many meaningful results, but the paper sometimes feels as if it is moving between constructs and levels of analysis without a single explicit organizing framework. In my view, the manuscript would become much stronger if the authors made the story more explicit: first establish the behavioural effect to the “breaking” trial and subsequent recovery in the repeated trust game, then evaluate whether the mechanism is emotional, perceptual, or strategic, and finally discuss what the absence of transfer to the repeated prisoner’s dilemma implies.

Major comments

1. The manuscript needs sharper conceptual precision about what the repeated trust game is measuring when participants act as trustees. A central conceptual issue appears very early in the paper. The manuscript often speaks in terms of “trust,” “repair of trust,” and “cooperation,” but in this task participants are not investors; they are trustees. This matters because the trustee role does not primarily measure willingness to trust under uncertainty. Rather, it measures how a participant responds to another’s trust or withdrawal of trust, which is closer to reciprocity, responsive cooperation, or repair-oriented prosocial responding.

I think the authors should clarify this more carefully in the introduction and discussion. At present, the manuscript sometimes slides between “trust,” “cooperation,” and “repair,” as if these were interchangeable. In the trust game, the trustee role is reactive and normatively reciprocal: “you trusted me, how do I respond?” In contrast, the repeated prisoner’s dilemma requires proactive strategic choice under simultaneous uncertainty: “what do I think the other will do, and should I cooperate now?”. Because of this, the null transfer to the prisoner’s dilemma may not simply indicate lack of generalization. It may indicate that the two tasks measure partially different constructs.

That distinction would greatly improve the paper’s conceptual coherence (e.g., Doppelhofer et al., 2025. doi. 10.1186/s40479-025-00295-2)

Therefore, failure to transfer may indicate that the intervention affects response to a perceived violation by a trusting partner, but not anticipatory cooperation in a simultaneous strategic environment. The question is then: which one of these constructs generate a symptoms (in BPD) that is specifically targeted by DBT?

Also, because the prisoner’s dilemma is introduced as a transfer task, the reader would benefit from a simple schematic figure comparing one trial of the trust game and one trial of the prisoner’s dilemma, highlighting the different decision structures. This would make the distinct psychological demands much easier to grasp. Maybe mirroring the role of the participants on two sides of the interaction in the two games.

2. The theoretical grounding in DBT is currently underspecified. Following my previous comment, the paper states that the intervention is “DBT-inspired,” but it is not yet clear which DBT process or module is actually being implemented. At several points the manuscript invokes distress tolerance and “opposite action,” but the intervention itself looks partly like psychoeducation about long-term consequences, partly like discouraging retaliation, and partly like explicit instruction toward a more prosocial strategy. That is not necessarily a problem, but the paper should be more precise about it.

The authors should clarify what level of processing is the intervention supposed to act on? The possibilities are not equivalent. The intervention could affect:

- perception/appraisal, for example by changing how a low investment is interpreted.
- emotion, for example by reducing anger, hurt, or arousal in response to the low investment.
- behaviour, for example by making participants inhibit retaliation despite preserved negative affect.

For how much I remember, DBT reduces impulsivity by acting simultaneously on perception (mindfulness), emotional reactivity (emotion regulation), and behavioural output (skills substitution), but how are you doing so?

Including the ratings of cooperativeness, selfishness, trustworthiness, and friendliness of the investors are potentially useful indicators of perceived partner intent. These could help address whether the intervention changes behaviour by changing the interpretation of the partner, even if emotion ratings do not change.

Further, the authors note that part of the effect might be attributable to the repeated emotional self-monitoring during the task but what if it’s the opposite? That is, if repeatedly asking about emotional status might trigger mindful strategies, what if repeatedly asking about the investment magnitude made them greedier? The subjective ratings of investment in Figure 1.B increases significantly over time, why do you think that happens? (Also clarify what is the “investment” rating).

At present the paper seems to conclude that because emotion ratings did not change, the intervention changed strategic behaviour rather than emotional reactivity. That is plausible, but it needs to be framed more carefully. It may be that the intervention altered the mapping from emotion to action rather than emotion per se. In other words, people may still have felt negative affect but behaved differently.

3. The programmed low-investment trial is the conceptual centrepiece, but the analysis and narrative do not foreground it enough. To me, the key theoretical event in the design is the pre-programmed low-investment round, because that is the moment where “trust withdrawal,” “violation,” or “rupture” is instantiated. The strongest result in the paper is not merely that the intervention group returned more overall (that currently is the first result). It is that the intervention group behaved differently at the critical violation trial and immediately after it (that is what you did your a-priori power analyses on). The authors do not need to redo the power analysis, but they should explain more clearly how the power logic relates to the final primary test. Out of curiosity, is there a significant effect of group (intervention vs control between) in the pre-post rupture behaviour, as hypothesised by the a-priori sample size calculation?

The event-study analysis seems to capture exactly this and, in my view, should be made much more central. Right now, the manuscript begins with overall mixed-effects results and only later arrives at the temporal dynamics around the low-investment event. I would suggest reversing the emphasis. The paper could first show that the intervention alters behaviour specifically at the rupture point and then show that this produces a broader shift in post-manipulation average return patterns.

Relatedly, I think the manuscript should discuss more explicitly why the low-investment trial is behaviourally meaningful in context. The rationale for placing it late in the round sequence is presumably that trust/cooperation has had time to build, so the low investment can be experienced as a breach relative to prior expectations. If so, this should be explained directly. Otherwise, readers may wonder why the “surprise” or “violation” value of the low investment is assumed, especially if the agent’s latent state already allows low investments. But maybe that was the “round #” predictor should do…

A useful addition would be an analysis or at least a discussion of change relative to recent history, not only absolute investment amount. For example:

- how much does the current investment deviate from the previous one?
- how much does it deviate from the recent average of prior rounds?
- is behaviour better predicted by absolute investment or by the drop from prior expectation?

A low investment after a series of high investments is a stronger violation than a low investment after already low or inconsistent investments (e.g., Palminteri et al., 2015. Doi. 10.1038/ncomms9096). Since the authors’ theoretical language emphasizes rupture and repair, a relative-deviation framing would be very helpful.

Probably would be helpful also to clarify what exactly means “low investment”. Was it fixed? How does it relate to the investor state? Is it possible to quantify it?

I’m wondering whether there are other ways of plotting the behaviour over time. Maybe the within subject difference (pre-post) of behaviour in the two groups over time? Interestingly even after the “rupture” the %return was above 30%, meaning that they never really penalised the investor. How many subjects (%) adopted a retaliatory strategy (<30%) before and after in the two groups?

4. The analytic plan needs a clearer organizing logic. Probably as my review does. The manuscript contains several models and several distinct kinds of findings, but the reader is not always told why each analysis comes when it does or what inferential role it plays. I think the paper would benefit greatly from a more explicit “analysis roadmap.”

For example, the authors could state something like:

1. We first test whether the effect is specifically concentrated around the programmed trust-withdrawal event.

2. We then evaluated whether a broader change in behaviour happens following the intervention

Regarding the LMM, I see that the mathematical description is more conceptually correct but sometimes the R annotation is more intuitive e.g.,

% return ~ condition * game * investment + round # + defection round + (1+ game * investment | subject)

Incidentally, is the distribution of the percentage return an issue for the LMM?

3. We then examine whether the intervention operates through changes in emotional responding, social perception, or strategic behaviour.

4. We then test transfer to a distinct but related game. Here I’d expect the same model (anova, LMM etc) you adopted to test the main objective in the first game.

5. Finally, we use HMMs to characterize latent behavioural states and heterogeneity in response.

That would make the methods, results, and discussion hang together much more naturally. Sometimes ven adding a simple line explaining the results (“this means that…”) helps the reader in keeping track of where the paper is heading. I know it’s sound trivial but might be really useful for some readers that are not native English speakers or clinician not familiar with the methods (and I am both!).

At present, some analyses feel under-motivated or insufficiently introduced. For instance, the reciprocity slopes (Figure 2.E) become important in the results, but their conceptual meaning is not fully introduced in the methods. Incidentally in Figure 2.D, why is the mean investment lower in the pre-intervention timepoint for the control group? How is this controlled for in the other analyses?

The relationship among emotion ratings, investor ratings, questionnaire moderators, and behavioural outputs is not framed as part of a unified mechanistic chain. Yet the paper implicitly points toward such a chain: investment/perceived partner behaviour → subjective appraisal/emotion → behavioural response.

The authors do not need a fully formal mediation model, but even a schematic version of this logic would help.

5. The questionnaire section feels underdeveloped relative to its importance. The manuscript includes BPD traits, emotion regulation difficulties, and reflective functioning questionnaires, which are highly relevant given the theoretical background. However, the results are summarized briefly as showing no moderation, and then the manuscript largely moves on.

Maybe clarifying what was expected could also point at which analyses perform. For example, if BPD traits were expected to moderate the intervention effect, was the relevant hypothesis that people with higher BPD traits would (e.g., Preti et al., 2023. doi. 10.1007/s11920-023-01468-y):

- retaliate more strongly after low investment?
- show a steeper drop in post-violation cooperation?
- benefit less from the intervention?
- show greater emotional reactivity to low investments?

A more nuanced question might be whether BPD traits moderate specific parts of the process. For example, does emotional reactivity over time vary as a function of BPD traits even if mean returns do not? Do BPD traits moderate the relationship between perceived selfishness of the investor and subsequent return? Do they predict which participants become stuck in low-return HMM states after the breaking trial?

Right now, the manuscript invokes BPD theory strongly, but the moderation findings are too limited to support some of the later inferences.

Would also be useful to spell out all the variables in the methods section (or maybe in Figure 1.A?) across three levels (individual, pre-post, trial). There are a lot of measures I couldn’t track over the manuscript. For example, how so speed and magnitude become a single rating named “investment”? Where are the 4 ratings of the investor? Which are the results within the intervention session (i.e., which were their behavioural responses during the intervention? And how many would have changed the strategy?). Also, what is the association between their actual change (behavioural response to rupture pre vs. post intervention) and declared change (“I’d change strategy” during the intervention)? When asked explicitly, which was the strategy chosen? Probably would be helpful to have the descriptive statistics of all the variables in the supplementary materials.

6. The HMM results are interesting but might benefit of a clearer. The HMM analysis is potentially one of the paper’s most original contributions, but it is not always easy to interpret. Several readers (again, me!) may struggle to understand what it means, for example, that a participant was in “state 1” or “state 3” on a given round, or why a shift in posterior occupancy matters beyond simply saying that mean returns were lower.

The authors should unpack this more explicitly in the Results section, not only in the Discussion. In particular, I would recommend:

- clearly explaining that the states correspond to distinct return policies or behavioural modes.
- explicitly stating what it means behaviourally to move toward lower versus higher states.

If possible, the figure legend and text should say something concrete like: “The intervention reduced the probability of occupying the most retaliatory low-return state after a trust withdrawal.” That is more interpretable than saying participants were less likely to get “stuck” without first explaining the meaning of the latent states.

Minor comments

1. The repeated prisoner’s dilemma results need at least one behavioural plot. Even if the task is not powered for subtle effects, a plot of cooperation across rounds or before/after the programmed defection would help readers see the null effect rather than only read a model summary. Connecting to the previous note on “how surprising” the violation is here, would be interesting to see how much a “defect” trial at trial 4, would affect the general trend.

2. The wording around “core behavioural strategy” may be too strong. At a few points, the discussion sounds as if the intervention clearly taught a core DBT strategy. Given the brevity and explicitness of the intervention, I would tone this down and describe it more conservatively as a brief DBT-inspired psychoeducational strategy or anti-retaliation prompt. I agree that is possible that part of the effect is due to the fact that you are explicitly telling them: don’t be nasty. Would be interesting how much that translate to clinical samples (BPD) but also actual interpersonal scenarios (De Panfilis et al., 2025, doi. 10.3390/brainsci15050530)

3. Strengthen the discussion! A big part of the discussion is structured as rebuttals to possible criticisms rather than explanations of why the observed pattern emerged. Both are useful, but the explanatory account should come first

# Reviewer report: Round 2, Reviewer A

**DOI:** 10.5334/cpsy.178.r3

**Authors:** Reviewer: A

## Recommendation:

Accept with Minor Revision

## Comments/Explanation (required). Please include references to any minor or major revisions required, as well as addressing any issues with language clarity. Authors will see these comments:

I thank the authors for their revisions. The flow of the results section has strongly improved, and the link to the figures is clearer. I also think the instruction effect is now fairly discussed. My only remaining recommendation is to plot the parameter recovery results. Currently, the authors only describe the results, but actual plots would provide much more useful information.

# Reviewer report: Round 2, Reviewer B

**DOI:** 10.5334/cpsy.178.r4

**Authors:** Reviewer: B

## Recommendation:

Accept (No Revisions Necessary)

## Comments/Explanation (required). Please include references to any minor or major revisions required, as well as addressing any issues with language clarity. Authors will see these comments:

The authors have addressed all of my comments satisfactorily. The revisions have substantially improved the clarity and readability of the manuscript, while further highlighting the importance and significance of the project. I believe the manuscript is now suitable for publication.

# Reviewer report: Round 1, Author Response

**DOI:** 10.5334/cpsy.178.r5

**Authors:** Rasmus Bruckner, Paolo Ossola Ossola

**Manuscript:** *(Re)building cooperation: Effects of a cognitive intervention on cooperative behaviour in games* (Computational Psychiatry).

## Summary of major changes

We thank the Editor-in-Chief and both reviewers for a careful and constructive read. A few themes ran through the reviews: clearer structure, sharper constructs, a fuller and validated hidden Markov model, and a more careful account of what a brief, explicit intervention can show. We revised on all of them. The main changes are:

1. **Structure.** The Results, the Methods roadmap, and the Discussion now follow one explicit organising logic: the response at the trust-withdrawal trial leads, then the broader post-manipulation shift, then mechanism (emotion, appraisal, strategy), then transfer to the Prisoner’s Dilemma, then latent-state heterogeneity. Every results paragraph is motivated in one line, tied to a figure panel, and closed with a plain “what this means” sentence.
2. **Construct precision.** We distinguish the trustee role (reactive, repair-oriented reciprocity) from trusting under uncertainty throughout the Introduction and Discussion, and carry the distinction into the Prisoner’s Dilemma interpretation.
3. **HMM exposition and validation.** A compact formal HMM specification now sits in the main Methods. A new Supplement section reports a posterior predictive check and a parameter-recovery analysis, and keeps interpretation at the level of state return-policies and occupancy, without reading the raw parameters.
4. **Instruction effect.** We withdraw the earlier claim that the design rules out an instruction effect, concede that the prompt is explicit and directive, and reframe the contribution around the behavioural specificity of the change.
5. **New analyses.** We add an event-study at the low investment, a relative-deviation analysis, a retaliator analysis, an investor-appraisal model, the within-intervention declared-change analysis, and trait moderation of low-return HMM-state membership.
6. **Tone.** We replace “core behavioural strategy” and similar phrasings with “brief, DBT-inspired psychoeducational (anti-retaliation) prompt.”

Point-by-point responses follow. Reviewer comments are quoted in full, each followed by our Response and the specific **Change** made.

**Reviewer 1**

Recommendation: Revisions Required. “In general, the manuscript seems to fit well with the Computational Psychiatry Journal… Despite the overall positive impression, I have several major concerns.”

We are grateful for the close engagement with both the exposition and the modelling.

## Reviewer A:

R1-1. Results description is hard to follow

Reviewer Feedback.

“*I found it difficult to follow the results during my first pass through the manuscript. One issue was that the figure subpanels are in a different order than the results description (e.g., first 2.A, then 2.C., but 2.B is mentioned much later). Also, in some paragraphs, there are no links to the figures (e.g., lines 245–247, 253–258). To address this, paragraphs on one type of analysis should be merged. For example, on page 8, paragraphs 2 and 3 both concern the same type of analysis. Paragraph 2 describes the main effect of condition, paragraph 3 describes the respective post-hoc contrasts. Currently, only paragraph 3 refers to Fig. 2.C. In other cases, analyses are not plotted or statistically quantified. Another example is paragraph 8 and 9 (page 8), where the authors could add figure panels, and the last paragraph, where the authors should add relevant statistics*.

*Finally, in some cases, the paragraphs (such as lines 271–272) are quite short, and the motivation behind the analyses is initially somewhat unclear. I suggest that the authors walk the readers more explicitly through the results, with a short motivation behind the analyses and their implications for the hypotheses*.”

## Author’s response

**Response.** We agree, and have rebuilt the Results around a single explicit organising logic. Each analysis now states the question it answers at the outset, points to the figure panel that bears on it, and ends by saying plainly what the result means. We merged paragraphs that report the same analysis, and we reordered Figure 2’s panels to follow the text.

**Change.** (1) Results reordered: effect at the trust-withdrawal trial → broader post-phase shift → mechanism (emotion/appraisal/strategy) → transfer → latent-state heterogeneity. (2) Figure 2 panels reordered to match, and Figure 1’s panels likewise reordered (now A design, B investor policy, C investor transitions, D intervention slide) so its references also run A→B→C→D; every results paragraph now references its panel. (3) Main-effect and post-hoc paragraphs merged. (4) Event-study promoted to a main-text figure (Figure 2.A); the questionnaire paragraph in the main text reports the moderation result (mostly null, with two exploratory RFQ-8 interactions) and points to Supplement Section 5, which reports the full ANOVA tables (F/df/p per moderator).

## Reviewer A:

R1-2. HMM background insufficiently introduced

**Reviewer Feedback**.

“*The general background of HMMs is very briefly introduced. For example, transition and emission parameters are insufficiently explained and defined. Another example is that the concept of a latent state, in particular with respect to the current task, is only superficially introduced. The authors should certainly refer to Figure 1.C and 1.D in the HMM results section as well (page 11), not just in the methods. Therefore, if the goal is that readers with a background in clinical psychology and not primarily computational modeling can smoothly follow the manuscript, then this section needs careful revision. On the other hand, also readers who want to understand the details of the model don’t find the relevant information in the main manuscript. The Methods currently don’t formally introduce the model; this is completely outsourced to the supplement*.”

## Author’s response

**Response.** Agreed. We have brought a compact, formal-but-accessible HMM specification into the main Methods and rewritten it to be readable by a clinical audience while still giving modellers the equations. The latent state is now explicitly defined as a behavioural mode specific to this task (a return-policy regime), with the emission distribution (state-conditional distribution over proportional returns) and the transition function (how the received investment moves a participant between modes) defined in words and notation.

**Change.** New Methods subsection formally defining the participant-return HMM (emission as a discretised Gaussian over feasible proportional returns; multinomial-logistic transition in investment and the condition/game contrast codes), reusing the Supplement equations; “latent state” defined in task terms; HMM Results now bridges to the investor HMM (Figure 1.B and 1.C) and the participant-state policies (Figure 4). Supplement Section 11 retains the extended derivation and per-model contrast coding.

## Reviewer A:

R1-3. HMM validation missing

**Reviewer Feedback**.

“*[…] Moreover, the manuscript currently lacks sufficient HMM validation analyses. I could not find a proper posterior predictive check indicating how well the model captures participant behavior in general. Does the model capture the behavior shown in Fig. 2.A? Would it be able to reproduce Fig. 2.C?*

*Moreover, I was wondering whether parameters can be accurately recovered, and suggest that the authors add a systematic parameter recovery analysis*.”

## Author’s response

**Response.** We thank the reviewer for pressing on validation, and have done both. (i) Posterior predictive check: we simulated 200 datasets from the fitted six-state HMM-full model and compared simulated to observed mean percentage return, round by round within each Condition and Game cell and for the four Condition by Game means (the round-by-round comparison addresses the reviewer’s question about Figure 2.A, the four means the question about Figure 2.C). The model reproduces the observed behaviour well (mean absolute error 0.014 across the 60 round-level cells, with 51 of 60 inside the 95% predictive interval; 0.005 for the four Condition by Game means, three of four inside the interval). (ii) Recovery analysis: simulating from the fitted model and refitting (100 replicates), the quantities we actually interpret recover well, namely the ordered state return-policies (expected-return mean absolute error 0.012) and the decoded post-withdrawal state occupancy (the lowest-return-state occupancy values reported in the text recover to within about one percentage point); the raw latent parameters and several individual transition probabilities are only weakly identified, as is expected for a six-state model with covariate-dependent transitions. We keep six states because the Bayesian Information Criterion selects that number as the best fit-versus-complexity trade-off, and because the quantities we interpret (the ordered return policies and the decoded occupancy) recover well at six states. The weak identifiability of the raw coefficients is a separate matter. It arises from label switching and a multimodal, weaklycurved likelihood (Supplement Section 11.2). That is precisely why we present the HMM descriptively, at the level of state return-policies and occupancy, and do not interpret individual emission or transition parameters as precise estimates.

**Change.** New Supplement subsection (Section 11.2) “Validation: posterior predictive check and recovery” with a figure of observed vs. simulated round-by-round means (Figure S7) and the error/coverage metrics, plus the recovery results; a three-sentence calibrated statement added to the main HMM Results that scopes interpretation to state returnpolicies and occupancy and disclaims parameter-level claims.

## Reviewer A:

R1-4. Demand / instruction effects

**Reviewer Feedback**.

“*On page 13 (lines 410–421), the authors argue that demand or instruction effects are unlikely due to (1) the way the intervention was framed, (2) the presence of individual differences, (3) the control condition, and (4) the absence of an effect in the prisoner’s dilemma*.

*In my opinion, all of these points can’t rule out an instruction effect. It is not unlikely that participants simply followed the instruction that clearly explained why cooperation makes sense in the task (Fig. 1.B). The presence of individual differences does not rule this out; virtually any task with clear instructions yields individual differences. Moreover, the control condition did not provide this instruction, and it is plausible that this straightforwardly explains why cooperative behavior was different between the conditions. Finally, the fact that the authors did not find a similar effect in the prisoner’s dilemma can be interpreted in terms of a quite specific instruction effect in the RTG rather than a general insight that participants should behave cooperatively across all kinds of situations in their daily life. Therefore, I think that the authors should revise their discussion of the possibility of instruction effects and what this would imply for generalizable effects of DBT interventions*.”

## Author’s response

**Response.** We accept this critique. Our earlier framing implied the design could rule out an instruction effect, which it cannot. We have rewritten this part of the Discussion to concede that the intervention contains an explicit, directive psychoeducational component, and to reframe the contribution accordingly. A brief, explicit, DBT-inspired anti-retaliation prompt produces a behaviourally specific change: returns rise across the post-manipulation game and diverge most sharply from the control group at the trust-withdrawal trial; the change is absent in a structurally different game (the Prisoner’s Dilemma); and it occurs without a measurable change in self-reported emotional reactivity. This specificity is descriptive and does not by itself argue against an instruction effect: a precise prompt to refrain from retaliating in the Trust Game would predict this pattern, including the absence of transfer to a game the prompt never mentioned. Having said that, we believe an instructional component would not, in any case, empty the result. Directive instruction and the expectations it sets are active ingredients of evidence-based psychotherapy rather than nuisance: cognitivebehavioural therapies explicitly teach and rehearse target behaviours (Beck, 2011), and the expectation that a framed response will help contributes to outcome in its own right (Trachsel & Grosse Holtforth, 2019). Even an explicit prompt is taken up unevenly, so a prompt that moves some participants and not others is the realistic case rather than a confound. Under full experimental control, the study shows a change that is dissociated from self-reported affect and unevenly distributed across participants in a way pre-existing traits do not explain (the new HMM-state moderation analysis; see our response on the questionnaire moderation below). Because the manipulation is a single prompt that combines several components, the design speaks to a change in the mapping from emotion to action, a reappraisal of the strategic value of not retaliating, rather than to a DBT-specific mechanism; isolating the active component, and gauging how far it would generalise, is a question for a follow-up that varies the prompt’s components.

**Change.** The Discussion section on demand/instruction is rewritten, with none of the four rejected arguments reused as decisive. The contribution is reframed around behavioural specificity, a new explicit limitation on instruction-versus-mechanism is added, and claims about DBT generalisability are tempered. It notes that an explicit instructional component is consistent with how directive psychotherapies operate, where instruction is taught (Beck, 2011) and expectation contributes to outcome (Trachsel & Grosse Holtforth, 2019). The rewrite also distinguishes the trustee construct (reactive, repair-oriented reciprocity, as opposed to proactive cooperation) and recasts the effect as a change in the emotion-to-action mapping; emotional reactivity itself was unchanged. The three readings of the PD null are now ordered construct-difference first, then strategic-context-specificity, closing with an explicit acknowledgement that a task-specific instruction effect cannot be excluded by the present design.

## Reviewer C:

Body recommendation: “Accept with Minor Revision”; structured comments note the manuscript “would become much stronger if the authors made the story more explicit.”

We thank Reviewer 2 for a thorough and constructive review, and treat the requested new analyses as substantive, addressing them in full.

R2-G. General / structure

**Reviewer Feedback**.

“*The manuscript structure can be improved in terms of conceptual framework (introduction) and integration of the main findings into the very same coherent picture (discussion). The abstract is accurate but could emphasize the key mechanism more explicitly*.

*At present, there are many meaningful results, but the paper sometimes feels as if it is moving between constructs and levels of analysis without a single explicit organizing framework. In my view, the manuscript would become much stronger if the authors made the story more explicit: first establish the behavioural effect to the “breaking” trial and subsequent recovery in the repeated trust game, then evaluate whether the mechanism is emotional, perceptual, or strategic, and finally discuss what the absence of transfer to the repeated prisoner’s dilemma implies*.”

## Author’s response

**Response.** We have adopted exactly this structure as the organising logic of the paper (Methods roadmap, Results, and Discussion). The introduction now builds one explicit conceptual framework (what the trustee role measures and why a brief reappraisal prompt should act at the moment of withdrawal), and the abstract foregrounds both the effect and its proposed mechanism: a behaviourally specific shift, general across the post-manipulation game and sharpest at the trust withdrawal, that we read as a change in the mapping from emotion to action, with emotional reactivity itself holding steady.

**Change.** Introduction conceptual framework strengthened; explicit analysis roadmap added to the Methods; Results reordered low-investment-first; Discussion integrated and structured explanation-first. The abstract retains its construct framing within the 250-word limit, with the mechanism sentence tightened to foreground behavioural specificity; the introduction sharpens the trustee-role construct as reactive, repair-oriented reciprocity rather than trusting under uncertainty, and aligns the foreshadowed results with the low-investment-first analysis.

## Reviewer C:

R2-1. Conceptual precision: trustee role ≠ “trust”

**Reviewer Feedback**.

“*The manuscript needs sharper conceptual precision about what the repeated trust game is measuring when participants act as trustees. A central conceptual issue appears very early in the paper. The manuscript often speaks in terms of “trust,” “repair of trust,” and “cooperation,” but in this task participants are not investors; they are trustees. This matters because the trustee role does not primarily measure willingness to trust under uncertainty. Rather, it measures how a participant responds to another’s trust or withdrawal of trust, which is closer to reciprocity, responsive cooperation, or repair-oriented prosocial responding*.

*I think the authors should clarify this more carefully in the introduction and discussion. At present, the manuscript sometimes slides between “trust,” “cooperation,” and “repair,” as if these were interchangeable. In the trust game, the trustee role is reactive and normatively reciprocal: “you trusted me, how do I respond?” In contrast, the repeated prisoner’s dilemma requires proactive strategic choice under simultaneous uncertainty: “what do I think the other will do, and should I cooperate now?”. Because of this, the null transfer to the prisoner’s dilemma may not simply indicate lack of generalization. It may indicate that the two tasks measure partially different constructs*.

*That distinction would greatly improve the paper’s conceptual coherence (e.g., Doppelhofer et al., 2025, 10.1186/s40479-025-00295-2). Therefore, failure to transfer may indicate that the intervention affects response to a perceived violation by a trusting partner, but not anticipatory cooperation in a simultaneous strategic environment. The question is then: which one of these constructs generate a symptoms (in BPD) that is specifically targeted by DBT*?

*Also, because the prisoner’s dilemma is introduced as a transfer task, the reader would benefit from a simple schematic figure comparing one trial of the trust game and one trial of the prisoner’s dilemma, highlighting the different decision structures. This would make the distinct psychological demands much easier to grasp. Maybe mirroring the role of the participants on two sides of the interaction in the two games*.”

## Author’s response

**Response.** We are grateful for this conceptual point. We agree, and following the reviewer’s specific guidance to “clarify this more carefully in the introduction and discussion,” we have sharpened the distinction there. The Introduction and Discussion now state explicitly that, as *trustees*, participants are not exercising trust but *responding to another’s extended and then withdrawn trust* (repair-oriented reciprocity, or resistance to retaliation), and we have audited those two sections to remove loose interchangeable use of “trust,” “cooperation,” and “repair.” We carry this through to the PD interpretation: because the PD requires proactive, simultaneous strategic choice instead of a reactive reciprocal response, the absence of transfer is consistent with partially distinct constructs and need not mean a simple failure to generalise. We added the suggested schematic as part of a main-text figure (Figure 3, panels A and B), following the reviewer’s recommendation that it belongs there as a comprehension aid. It contrasts a single Trust Game trial with a single Repeated Prisoner’s Dilemma trial, with the two interacting roles shown on each side, on a common time axis that makes the sequential-versus-simultaneous structure explicit, and it shows the mechanistic chain. It is referenced from the transfer analysis and the Discussion. We also added a brief Discussion note linking the trustee/reciprocity construct to the BPD rupture-and-repair phenomenology that DBT’s distress-tolerance / opposite-action skills target.

**Change.** The trustee-versus-trust distinction is stated and the terminology audited within the **Introduction and Discussion**. The title and abstract keep the broader, accurate umbrella term “cooperative behaviour”; we confirmed the abstract uses “trust” only for the investor’s extended or withdrawn trust and for the violation, never to describe the trustee as trusting, so the title and abstract are unchanged. The PD interpretation is revised and Doppelhofer et al. 2025 is cited. A new conceptual schematic (one TG trial versus one PD trial, the two interacting roles shown on each side on a common time axis) is combined with a schematic of the mechanistic chain into the main-text figure (Figure 3, panels A and B), cross-referenced from the transfer result and the Discussion. A Discussion sentence links the trustee/reciprocity construct to BPD’s rupture-and-repair deficit and to DBT’s targeted skills.

## Reviewer C:

R2-2. DBT grounding underspecified

**Reviewer Feedback**.

“*[…] Following my previous comment, the paper states that the intervention is “DBT-inspired,” but it is not yet clear which DBT process or module is actually being implemented. At several points the manuscript invokes distress tolerance and “opposite action,” but the intervention itself looks partly like psychoeducation about long-term consequences, partly like discouraging retaliation, and partly like explicit instruction toward a more prosocial strategy. That is not necessarily a problem, but the paper should be more precise about it*.

*The authors should clarify what level of processing is the intervention supposed to act on? The possibilities are not equivalent. The intervention could affect*:

- *perception/appraisal, for example by changing how a low investment is interpreted*.
- *emotion, for example by reducing anger, hurt, or arousal in response to the low investment*.
- *behaviour, for example by making participants inhibit retaliation despite preserved negative affect*.

*For how much I remember, DBT reduces impulsivity by acting simultaneously on perception (mindfulness), emotional reactivity (emotion regulation), and behavioural output (skills substitution), but how are you doing so*?

*Including the ratings of cooperativeness, selfishness, trustworthiness, and friendliness of the investors are potentially useful indicators of perceived partner intent. These could help address whether the intervention changes behaviour by changing the interpretation of the partner, even if emotion ratings do not change*.

*Further, the authors note that part of the effect might be attributable to the repeated emotional self-monitoring during the task but what if it’s the opposite? That is, if repeatedly asking about emotional status might trigger mindful strategies, what if repeatedly asking about the investment magnitude made them greedier? The subjective ratings of investment in Figure 1.B increases significantly over time, why do you think that happens? (Also clarify what is the “investment” rating)*.

*At present the paper seems to conclude that because emotion ratings did not change, the intervention changed strategic behaviour rather than emotional reactivity. That is plausible, but it needs to be framed more carefully. It may be that the intervention altered the mapping from emotion to action rather than emotion per se. In other words, people may still have felt negative affect but behaved differently*.”

## Author’s response

**Response.** We now specify the intervention as multi-component and predominantly an opposite-action-framed psychoeducation (acting contrary to the retaliatory urge), and we are explicit that it is directive, openly instructing participants against retaliation. We separate the levels: appraisal, emotion, behaviour. We added an analysis of the four investor ratings as an index of partner appraisal. No rating showed a Condition by Game interaction. The intervention group did rate the investor somewhat more cooperative and less selfish overall (a main effect of Condition), but this cannot account for the behavioural result, for two reasons. First, the effect we are explaining is a Condition by Game interaction: the divergence in returns appears only after the manipulation. An appraisal difference can produce that interaction only if it too varies by Condition and Game, which it does not. Second, the four investor ratings were collected retrospectively at the end of each game, so they cannot be a within-game cause of the returns; a more charitable overall appraisal is at least as plausibly a consequence of having returned more. The behavioural effect is therefore unlikely to run through a change in partner appraisal. We also analysed the control group’s grid ratings over rounds and clarified what they are (the speed-and-magnitude evaluation, not an emotion measure). The rating is veridical: it tracks the objective investment closely, so it rises over the game because the investments themselves rose, and once the objective investment is in the model the round trend is negligible. We therefore find no support for the reviewer’s reverse hypothesis that repeatedly rating investment magnitude made control participants more self-interested; the rating and the returns both follow the partner’s actual behaviour rather than a self-generated drift (we keep the grid-content confound as a stated limitation). We adopt the reviewer’s framing that the intervention most plausibly altered the emotion→action mapping while leaving emotional reactivity itself untouched.

**Change.** Methods: the intervention is now mapped to DBT’s distress-tolerance and emotion-regulation modules and to the opposite-action skill, delivered as brief psychoeducation rather than full skills practice, with the scope limitation stated explicitly. The “investment”/grid rating is defined as the speed-and-magnitude evaluation, not an emotion measure. New analyses: an investor-rating appraisal model (Mechanism section), and the control grid-rating trajectory and reverse-direction hypothesis (Supplement Section 9.5, “The control group’s per-round rating is veridical”). Discussion: the emotion-to-action-mapping reframe, with the explicit, directive nature of the intervention conceded as a limitation on mechanistic interpretation.

## Reviewer C:

R2-3. Foreground the programmed low-investment trial

**Reviewer Feedback**.

“*To me, the key theoretical event in the design is the pre-programmed low-investment round, because that is the moment where “trust withdrawal,” “violation,” or “rupture” is instantiated. The strongest result in the paper is not merely that the intervention group returned more overall (that currently is the first result). It is that the intervention group behaved differently at the critical violation trial and immediately after it (that is what you did your a-priori power analyses on). The authors do not need to redo the power analysis, but they should explain more clearly how the power logic relates to the final primary test. Out of curiosity, is there a significant effect of group (intervention vs control between) in the pre-post rupture behaviour, as hypothesised by the a-priori sample size calculation*?

*The event-study analysis seems to capture exactly this and, in my view, should be made much more central. Right now, the manuscript begins with overall mixed-effects results and only later arrives at the temporal dynamics around the low-investment event. I would suggest reversing the emphasis. The paper could first show that the intervention alters behaviour specifically at the rupture point and then show that this produces a broader shift in post-manipulation average return patterns*.

*Relatedly, I think the manuscript should discuss more explicitly why the low-investment trial is behaviourally meaningful in context. The rationale for placing it late in the round sequence is presumably that trust/cooperation has had time to build, so the low investment can be experienced as a breach relative to prior expectations. If so, this should be explained directly. Otherwise, readers may wonder why the “surprise” or “violation” value of the low investment is assumed, especially if the agent’s latent state already allows low investments. But maybe that was the “round #” predictor should do*…

*A useful addition would be an analysis or at least a discussion of change relative to recent history, not only absolute investment amount. For example*:

- *how much does the current investment deviate from the previous one*?
- *how much does it deviate from the recent average of prior rounds*?
- *is behaviour better predicted by absolute investment or by the drop from prior expectation*?

*A low investment after a series of high investments is a stronger violation than a low investment after already low or inconsistent investments (e.g., Palminteri et al., 2015, 10.1038/ncomms9096). Since the authors’ theoretical language emphasizes rupture and repair, a relative-deviation framing would be very helpful*.

*Probably would be helpful also to clarify what exactly means “low investment”. Was it fixed? How does it relate to the investor state? Is it possible to quantify it*?

*I’m wondering whether there are other ways of plotting the behaviour over time. Maybe the within subject difference (pre-post) of behaviour in the two groups over time? Interestingly even after the “rupture” the %return was above 30%, meaning that they never really penalised the investor. How many subjects (%) adopted a retaliatory strategy (<30%) before and after in the two groups?*”

## Author’s response

**Response.** This was a particularly helpful comment. We agree this is the central result and have restructured accordingly: the event-study around the programmed low-investment trial leads the Behavioural results, with the omnibus mixed model presented as the broader corroboration. We clarify how the a-priori power logic relates to the final primary test: the sample size was determined to detect a small within–between Condition×Game interaction in the 2×2 design, and the low-investment window is where that hypothesised interaction is expected to manifest (we are careful not to claim the low-investment-window subset was independently powered). We report the Condition×Game effect in the low-investment window directly. We added a relative-deviation analysis (return as a function of deviation from the previous investment and from the running mean, and a comparison of absolute-investment vs. deviation-from-expectation models), and we quantify “low investment” precisely relative to the investor’s latent state. That comparison shows returns track the absolute size of the investment; the deviation from recent rounds adds little (Supplement Section 9.3). This does not undercut the rupture framing. The programmed investment functions as a violation because two units is absolutely very low, and is sent while the agent is usually in a medium- or high-trust state (in a low-trust state on only 21% of these trials). It runs against the agent’s own disposition; deviation from a running average plays no part. We therefore anchor the “violation/rupture” language to this off-policy, absolutely-low character of the investment; the data give no support to a relative-deviation account. We added the within-subject pre–post difference plot (Figure 2.B) and report the proportion of participants adopting a retaliatory strategy (return <30%) before vs. after, by group, noting that mean returns remained above 30% (participants rarely fully punished).

**Change.** The Results were reordered so the event-study leads the Behavioural results. The Methods power-logic paragraph now clarifies that the omnibus Condition × Game is the powered primary test and that the low-investment-window event-study is a focused secondary analysis. The low-investment-window Condition × Game effect is reported in the main text (F(1, 1547.5) = 21.03, p < .001). We quantify the low investment relative to the agent’s latent state: a 2-unit value, about 82% below the preceding investment of 11.4 units, with the agent in a low-trust latent state on only 21% of low-investment trials. An absolute-investment versus deviation-from-expectation comparison is added (Supplement Section 9.3; the absolute investment dominates). A retaliator analysis is added to the main text and Supplement Section 9.4: the proportion with mean return below 30% at and after the low investment rose in the control group from 20.8% to 36.5% pre to post (McNemar χ²(1) = 14.05, p < .001) but stayed flat in the intervention group (17% to 20.1%, p = .51), robust across 25%, 30%, and 35% thresholds. The within-subject pre-minus-post difference plot was promoted to Figure 2.B. Palminteri et al. 2015 is cited.

## Author’s response

R2-4. Analytic plan needs a clearer organising logic

**Reviewer Feedback**.

“*[…] Probably as my review does. The manuscript contains several models and several distinct kinds of findings, but the reader is not always told why each analysis comes when it does or what inferential role it plays. I think the paper would benefit greatly from a more explicit “analysis roadmap*.”

*For example, the authors could state something like*:

1. *We first test whether the effect is specifically concentrated around the programmed trust-withdrawal event*.

2. *We then evaluated whether a broader change in behaviour happens following the intervention*.

*Regarding the LMM, I see that the mathematical description is more conceptually correct but sometimes the R annotation is more intuitive, e.g*.,

% return ~ condition * game * investment + round # + defection round + (1 + game * investment | subject)

*Incidentally, is the distribution of the percentage return an issue for the LMM*?

3. *We then examine whether the intervention operates through changes in emotional responding, social perception, or strategic behaviour*.

4. *We then test transfer to a distinct but related game. Here I’d expect the same model (ANOVA, LMM etc.) you adopted to test the main objective in the first game*.

5. *Finally, we use HMMs to characterize latent behavioural states and heterogeneity in response*.

*That would make the methods, results, and discussion hang together much more naturally. Sometimes even adding a simple line explaining the results (“this means that…”) helps the reader in keeping track of where the paper is heading. I know it sounds trivial but might be really useful for some readers that are not native English speakers or clinician not familiar with the methods (and I am both!)*.

*At present, some analyses feel under-motivated or insufficiently introduced. For instance, the reciprocity slopes (Figure 2.E) become important in the results, but their conceptual meaning is not fully introduced in the methods. Incidentally in Figure 2.D, why is the mean investment lower in the pre-intervention timepoint for the control group? How is this controlled for in the other analyses*?

*The relationship among emotion ratings, investor ratings, questionnaire moderators, and behavioural outputs is not framed as part of a unified mechanistic chain. Yet the paper implicitly points toward such a chain*:

*investment / perceived partner behaviour → subjective appraisal / emotion → behavioural response*.

*The authors do not need a fully formal mediation model, but even a schematic version of this logic would help*.”

## Author’s response

**Response.** We added the explicit roadmap (essentially the reviewer’s list) to the Methods and use it as the organising logic of Results and Discussion. We give the R-formula annotation alongside the equation. We address the distribution of percentage returns directly: we report distributional diagnostics and a robustness re-fit as a binomial mixed model appropriate for a bounded proportion; the key Condition by Game interaction holds there (z = 10.86, p < .001), so the conclusions do not depend on the Gaussian approximation. We define the reciprocity slope where the model is introduced. We explain the pre-phase investment difference between conditions: investments are generated by the adaptive HMM investor reacting to participants’ returns, so they are endogenous, which is precisely why investment enters the return model as a covariate; we also verify that returns did not differ between conditions before the manipulation (the pre-manipulation Intervention-versus-Control contrast is non-significant), so the post-manipulation divergence is not inherited from a pre-existing imbalance. We add a single schematic of the mechanistic chain, combined with a trust-game-versus-Prisoner’s-Dilemma trial comparison into one display item.

**Change.** Methods: the roadmap, the R-formula, the reciprocity-slope definition, and the LMM distributional justification are added. “This means…” sentences run throughout the Results. The investor-investment difference between conditions (originally Fig 2.D in the previous figure layout, now Supplement Figure S5 / Section 7) is explained in the Mechanism section as endogenous, since the adaptive investor reacts to participants’ returns, and is controlled for as a covariate in every return model. The mechanistic chain is drawn as a conceptual model of the trustee’s response (from the partner’s behaviour, through a latent appraisal of the partner and an emotional reaction, to the return) in the main-text Figure 3 (panel B), alongside the trust-game-versus-Prisoner’s-Dilemma trial comparison. The binomial robustness re-fit is in Supplement Section 9.2.

## Reviewer C:

R2-5. Questionnaire section underdeveloped

**Reviewer Feedback**.

“*The manuscript includes BPD traits, emotion regulation difficulties, and reflective functioning questionnaires, which are highly relevant given the theoretical background. However, the results are summarized briefly as showing no moderation, and then the manuscript largely moves on*.

*Maybe clarifying what was expected could also point at which analyses to perform. For example, if BPD traits were expected to moderate the intervention effect, was the relevant hypothesis that people with higher BPD traits would (e.g., Preti et al., 2023, 10.1007/s11920-023-01468-y)*:

- *retaliate more strongly after low investment*?
- *show a steeper drop in post-violation cooperation*?
- *benefit less from the intervention*?
- *show greater emotional reactivity to low investments*?

*A more nuanced question might be whether BPD traits moderate specific parts of the process. For example, does emotional reactivity over time vary as a function of BPD traits even if mean returns do not? Do BPD traits moderate the relationship between perceived selfishness of the investor and subsequent return? Do they predict which participants become stuck in low-return HMM states after the breaking trial*?

*Right now, the manuscript invokes BPD theory strongly, but the moderation findings are too limited to support some of the later inferences*.

*Would also be useful to spell out all the variables in the methods section (or maybe in Figure 1.A?) across three levels (individual, pre-post, trial). There are a lot of measures I couldn’t track over the manuscript. For example, how do speed and magnitude become a single rating named “investment”? Where are the 4 ratings of the investor? Which are the results within the intervention session (i.e., which were their behavioural responses during the intervention? And how many would have changed the strategy?). Also, what is the association between their actual change (behavioural response to rupture pre vs. post intervention) and declared change (“I’d change strategy” during the intervention)? When asked explicitly, which was the strategy chosen? Probably would be helpful to have the descriptive statistics of all the variables in the supplementary materials*.”

## Author’s response

**Response.** We agree the questionnaire treatment was thin relative to the theoretical weight we place on BPD. We now state the a-priori moderation hypotheses explicitly and test the more targeted questions the reviewer raises: whether BPD traits moderate emotional reactivity over time and the perceived-selfishness→return link, and whether the intervention itself (rather than questionnaire scores) predicts adoption of a post-low-investment retaliatory strategy. We add a variable table covering the individual and trial levels (with game-phase information described in the surrounding prose, since the design is balanced at that level). We clarify the grid (“investment” = speed×magnitude evaluation, defined in Methods) and point to where the four investor ratings are reported. We add an analysis of the within-intervention free-text responses (how participants said they would respond after reading the educational slide; the proportion declaring they would change strategy) and relate declared change to actual pre→post change in low-investment-window returns. Full descriptive statistics are added to the Supplement. Finally, on the reviewer’s broader point that the manuscript invoked BPD theory more strongly than the moderation findings support, we have tempered those inferences. The Introduction now states the trait expectations as a-priori and directional, not confirmed here. The Results report the moderation as mostly null and flag the two exploratory RFQ-8 interactions as hypothesis-generating rather than confirmed. The Discussion treats the borderline-personality relevance as a motivating rationale and an open question for clinical samples, not as something the present non-clinical data establish.

**Change.** Methods: a-priori hypotheses for the questionnaire moderators stated; Preti et al. 2023 cited; “investment”/grid rating defined. Supplement: variable table at individual and trial levels (Section 9.1); expanded moderation (Section 5 reports the result on mean returns, mostly null with two exploratory RFQ-8 interactions; Section 5.2 reports three targeted moderation tests, summarised below); new subsection on declared change and its relation to behaviour (Section 9.6). The main text summarises this in a short paragraph and points to the Supplement for the full tables.

Targeted moderation findings (Supplement Section 5.2):

- **(a) The trial-level coupling between investment received and immediately-reported emotion is trait-graded in a game-dependent way, with opposite directions for valence and arousal.** Here the slope measures how tightly a participant’s reported emotion tracked the investment received (the change in the grid rating per standardised unit of investment); a higher slope means a tighter coupling, and trait-graded means this slope differs across trait levels. In the post-manipulation game, the slope of valence on investment rose with DERS (32.0 at –1 SD vs 37.9 at +1 SD) and with PAI-BOR (32.6 vs 37.4), whereas pre-game slopes were uniform across trait levels (valence × DERS × Game F(1, 4516.5) = 8.25, p = .004; valence × PAI-BOR × Game F(1, 4514.9) = 4.27, p = .039). For arousal, the pre-game trait gradient (slope falling with DERS, from 31.9 at –1 SD to 22.0 at +1 SD) attenuated in the post-game (26.3 vs 24.6; arousal × DERS × Game F(1, 4502.0) = 15.72, p < .001). We report this as a descriptive, exploratory pattern and do not present it as evidence about mechanism: the analyses are exploratory and non-preregistered, the design does not include emotion ratings from the control group (so pre-to-post change in the intervention group is confounded with practice, fatigue, and pre-game low-investment carryover), and the 2D-grid self-report cannot adjudicate among several candidate accounts (heightened evaluative coupling under emotion-dysregulation conditions, response-style differences correlated with trait scores, or trait-modulated adaptation across rounds).
- **(b) Perceived investor selfishness lowers returns but is not moderated by PAI-BOR, DERS, or RFQ-8** (perceived selfishness F(1, 513.4) = 15.54, p < .001; perceived-selfishness × PAI-BOR F(1, 568.1) = 0.00, p > .99; × DERS F(1, 550.4) = 1.20, p = .27; × RFQ-8 F(1, 524.6) = 2.10, p = .15; no condition-dependent moderation either).
- **(c) For the reviewer’s specific question about who becomes “stuck in low-return HMM states”, we now test this directly on the latent states and corroborate it with a behavioural proxy.** Reusing the locally-decoded trustee states from the main-text HMM, we modelled whether trait scores predict occupying the lowest-return (most retaliatory) state on the trial after the post-manipulation low investment, the round at which the paper indexes getting stuck. None did (PAI-BOR OR = 1.05, p = .73; DERS OR = 1.08, p = .56; RFQ-8 OR = 0.95, p = .70, each adjusted for Condition). The intervention, by contrast, reduced the odds of being stuck there (OR = 0.40, p = .001). The behavioural proxy (retaliator = mean return < 30% at and after the low investment) gives the same answer (PAI-BOR OR = 0.94, p = .73; DERS 1.04, p = .82; RFQ-8 0.95, p = .79; intervention OR = 0.44, p = .001). The pattern the reviewer anticipated, that borderline traits predict getting stuck, does not hold in this non-clinical sample; here it is the manipulation that governs who gets stuck, while the trait measures do not. This is now reported in the main-text HMM Results and in Supplement Section 5.2.

The free-text coding for the declared-change analysis used a fixed codebook applied by a large language model in two independent passes, the second blind to the first (Cohen’s κ = .90, 95.6% agreement); a co-author adjudicated the seven disagreements (six decisively, one ambiguous reported under both resolutions). About 35% of participants (56 of 159) declared a shift toward cooperation, and declared change predicted the actual pre- to post-manipulation change in low-investment-window returns (Welch t(112.9) = -2.75, p = .007), an association that held under every resolution of the disagreements (largest p = .015).

## Reviewer C:

R2-6. HMM results need clearer interpretation

**Reviewer Feedback**.

“*The HMM analysis is potentially one of the paper’s most original contributions, but it is not always easy to interpret. Several readers (again, me!) may struggle to understand what it means, for example, that a participant was in “state 1” or “state 3” on a given round, or why a shift in posterior occupancy matters beyond simply saying that mean returns were lower*.

*The authors should unpack this more explicitly in the Results section, not only in the Discussion. In particular, I would recommend*:

- *clearly explaining that the states correspond to distinct return policies or behavioural modes*.
- *explicitly stating what it means behaviourally to move toward lower versus higher states*.

If possible, the figure legend and text should say something concrete like: ”The intervention reduced the probability of occupying the most retaliatory low-return state after a trust withdrawal.”* That is more interpretable than saying participants were less likely to get “stuck” without first explaining the meaning of the latent states.”*

## Author’s response

**Response.** We rewrote the HMM Results in plain behavioural language. We state that each state is a distinct returnpolicy regime, order states by expected return, and describe what moving to a lower vs. higher state means (more vs. less retaliatory/withholding). We adopt the reviewer’s concrete phrasing in both the text and the figure legend, anchored to the actual numbers (e.g., post-low-investment occupancy of the lowest-return state at round 14: 15% intervention vs 31% control; 17% of intervention participants nonetheless remained in the lowest-return state directly after the post-manipulation low investment, i.e., non-responders).

**Change.** HMM Results rewritten (states as behavioural modes; directional meaning; concrete sentence anchored to specific occupancy numbers); Figure 4 legend made concrete. No new analysis (numbers already estimated).

## Reviewer C:

R2-m1. RPD needs a behavioural plot

**Reviewer Feedback**.

“***The repeated prisoner’s dilemma results need at least one behavioural plot.** Even if the task is not powered for subtle effects, a plot of cooperation across rounds or before/after the programmed defection would help readers see the null effect rather than only read a model summary. Connecting to the previous note on “how surprising” the violation is here, would be interesting to see how much a “defect” trial at trial 4 would affect the general trend*.”

## Author’s response

**Response.** Added. A cooperation-rate-by-round plot for the RPD, marking the round-4 programmed defection, is now in the main text; it shows the post-defection drop and the absence of a condition difference.

**Change.** RPD cooperation-by-round figure promoted to a main-text panel (Figure 2.D), with the round-4 programmed defection marked.

## Reviewer C:

R2-m2. “core behavioural strategy” is too strong

**Reviewer Feedback**.

“***The wording around “core behavioural strategy” may be too strong.** At a few points, the discussion sounds as if the intervention clearly taught a core DBT strategy. Given the brevity and explicitness of the intervention, I would tone this down and describe it more conservatively as a brief DBT-inspired psychoeducational strategy or anti-retaliation prompt. I agree that is possible that part of the effect is due to the fact that you are explicitly telling them: don’t be nasty. Would be interesting how much that translates to clinical samples (BPD) but also actual interpersonal scenarios (De Panfilis et al., 2025, 10.3390/brainsci15050530)*.”

## Author’s response

**Response.** Agreed, and we concede the reviewer’s point that part of the effect may follow from explicitly telling participants not to retaliate. We replaced “core behavioural strategy” and similar phrasings with “brief, DBT-inspired psychoeducational (anti-retaliation) prompt,” and added a measured note on what would be needed to translate to clinical (BPD) and naturalistic interpersonal contexts.

**Change.** Terminology toned down throughout Discussion (“brief, explicit, DBT-inspired prompt” in place of “core behavioural strategy”); a translation sentence in the Limitations paragraph gestures toward clinical (BPD) and naturalistic interpersonal contexts; De Panfilis et al. 2025 cited.

## Reviewer C:

R2-m3. Strengthen the Discussion (explanation before rebuttal)

**Reviewer Feedback**.

“***Strengthen the discussion.** A big part of the discussion is structured as rebuttals to possible criticisms rather than explanations of why the observed pattern emerged. Both are useful, but the explanatory account should come first*.”

## Author’s response

**Response.** We restructured the Discussion to lead with a positive, integrated explanatory account (why a brief reappraisal prompt produces a behavioural shift that is general across the post-manipulation game, sharpest at the trust withdrawal, not mediated by emotional reactivity, and absent in the Prisoner’s Dilemma), and only then address alternative explanations, including an explicit concession that the present design cannot separate an instruction effect from a DBT mechanism.

**Change.** Discussion reordered: explanatory account first, alternatives second; integrated with the trustee-construct distinction and the instruction-effect concession.

# Reviewer report: Round 2, Author Response

**DOI:** 10.5334/cpsy.178.r6

**Authors:** Rasmus Bruckner, Paolo Ossola Ossola

**Manuscript:** *(Re)building cooperation: Effects of a cognitive intervention on cooperative behaviour in games* (Computational Psychiatry).

Dear Dr Diaconescu and Reviewers,

We thank the Editor and Reviewers for the positive assessment of our revised manuscript. We have addressed the sole remaining recommendation as described below.

## Reviewer A:

Comment

“*My only remaining recommendation is to plot the parameter recovery results. Currently, the authors only describe the results, but actual plots would provide much more useful information*.”

## Author’s response

**Response.** We thank the reviewer for this helpful recommendation. We agree that displaying the recovery results makes the model validation clearer and allows readers to assess the correspondence between the generating and recovered quantities directly. We have therefore added a new four-panel recovery figure based on the existing 100 simulated datasets. The figure shows recovery of the state return policies, decoded state occupancy, transition probabilities, and the correspondence between generating and recovered raw coefficients.

**Change.** Supplementary Information, Section 11.2, Figure S8 (page 18). No other manuscript text or analyses were changed.

## Reviewer B:

Comment

“*The authors have addressed all of my comments satisfactorily. The revisions have substantially improved the clarity and readability of the manuscript, while further highlighting the importance and significance of the project. I believe the manuscript is now suitable for publication*.”

## Author’s response

**Response.** We thank the reviewer for their positive assessment and for confirming that the previous revisions satisfactorily addressed their comments. No additional revisions were requested.

We thank the Editor and Reviewers again for their careful and constructive evaluation of our manuscript.
